# Differences in the characteristics and pulmonary toxicity of nano- and micron-sized respirable coal dust

**DOI:** 10.1186/s12931-022-02120-8

**Published:** 2022-07-30

**Authors:** Yinci Zhang, Amin Li, Jiafeng Gao, Jiaojiao Liang, Niandie Cao, Shuping Zhou, Xiaolong Tang

**Affiliations:** 1 Medical School, Anhui University of Science & Technology, Huainan, 232001 China; 2 Institute of Environment-Friendly Materials and Occupational Health of Anhui University of Science and Technology, Wuhu, 241003 China; 3 First Affiliated Hospital, Anhui University of Science & Technology, Huainan, 232001 China

**Keywords:** Coal dust nanoparticles, Coal dust micron particles, Coal worker’s pneumoconiosis, Pulmonary fibrosis, Epithelial–mesenchymal transition, Acute pulmonary toxicity

## Abstract

**Background:**

The characteristics of coal dust (CD) particles affect the inhalation of CD, which causes coal worker’s pneumoconiosis (CWP). CD nanoparticles (CD-NPs, < 500 nm) and micron particles (CD-MPs, < 5 μm) are components of the respirable CD. However, the differences in physicochemical properties and pulmonary toxicity between CD-NPs and CD-MPs remain unclear.

**Methods:**

CD was analyzed by scanning electron microscopy, Malvern nanoparticle size potentiometer, energy dispersive spectroscopy, infrared spectroscopy, and electron paramagnetic resonance spectroscopy. CCK-8 assay, ELISA, transmission electron microscope, JC-1 staining, reactive oxygen species activity probe, calcium ion fluorescent probe, AO/EB staining, flow cytometry, and western blot were used to determine the differences between CD-NPs and CD-MPs on acute pulmonary toxicity. CCK-8, scratch healing and Transwell assay, hematoxylin–eosin and Masson staining, immunohistochemistry, immunofluorescence, and western blot were applied to examine the effects of CD-NPs and CD-MPs on pneumoconiosis.

**Results:**

Analysis of the size distribution of CD revealed that the samples had been size segregated. The carbon content of CD-NPs was greater than that of CD-MPs, and the oxygen, aluminum, and silicon contents were less. In in vitro experiments with A549 and BEAS-2B cells, CD-NPs, compared with CD-MPs, had more inflammatory vacuoles, release of pro-inflammatory cytokines (IL-6, IL-1β, TNFα) and profibrotic cytokines (CXCL2, TGFβ1), mitochondrial damage (reactive oxygen species and Ca^2+^ levels and decreased mitochondrial membrane potential), and cell death (apoptosis, pyroptosis, and necrosis). CD-NPs-induced fibrosis model cells had stronger proliferation, migration, and invasion than did CD-MPs. In in vivo experiments, lung coefficient, alveolar inflammation score, and lung tissue fibrosis score (mean: 1.1%, 1.33, 1.33) of CD-NPs were higher than those of CD-MPs (mean: 1.3%, 2.67, 2.67). CD-NPs accelerated the progression of pulmonary fibrosis by upregulating the expression of pro-fibrotic proteins and promoting epithelial–mesenchymal transition. The regulatory molecules involved were E-cadherin, N-cadherin, COL-1, COL-3, ZO-1, ZEB1, Slug, α-SMA, TGFβ1, and Vimentin.

**Conclusions:**

Stimulation with CD-NPs resulted in more pronounced acute and chronic lung toxicity than did stimulation with CD-MPs. These effects included acute inflammatory response, mitochondrial damage, pyroptosis, and necrosis, and more pulmonary fibrosis induced by epithelial–mesenchymal transition.

**Supplementary Information:**

The online version contains supplementary material available at 10.1186/s12931-022-02120-8.

## Introduction

Coal mining and processing generate dusts, among which coal dust (CD) is one of the main health hazards for coal workers [1]. The elemental composition of CD particles varies greatly in various regions [2]. Therefore, in this study, we compared the differences in physicochemical properties, the acute pulmonary toxicity, and the chronic pulmonary fibrosis-inducing ability of CD-nanoparticles (CD-NPs) and CD-micron particles (CD-MPs) and their associated molecular mechanisms, focusing on potential adverse effects of CD size on the lung.

Nanoparticles have been found more toxic than larger particles due to their large surface area and easier absorption by organisms [3, 4]. Sarver [5] and Keles [6] et al. determined the particle size distribution of 171 sets of respirable CD samples collected from 25 underground coal mines in several regions of the United States, showing the presence of CD-NPs (< 500 nm) in respirable CD. CD particles can impair normal lung function in humans [7], increase the risk of pneumoconiosis [8], and damage alveoli in rats [9]. Therefore, it is reasonable to infer that CD-NPs have more pulmonary toxicity and are more conducive to coal worker’s pneumoconiosis (CWP) than CD-MPs. However, there are few studies on the effects of CD particles of various size on human pulmonary health.

Early studies showed that chronic inflammation is the root cause of pulmonary fibrosis, and subsequent studies have found that the pathogenesis of pulmonary fibrosis is the disruption of lung homeostasis by foreign substances, resulting in irreversible damage to alveolar epithelial cells and lung epithelial cells (mitochondrial damage, apoptosis, focal death, and necrosis) and mesenchymal phenotype change, which in turn promotes lung fibrosis [10–12]. Among alveolar epithelial cells and lung epithelial cells, impairment of normal functional capacity and development of a profibrotic phenotype contribute to the development of idiopathic pulmonary fibrosis [13]. Epithelial–mesenchymal transition (EMT) is an important factor in pulmonary fibrosis, contributing to the activation of myofibroblasts and the production of the extracellular matrix [14–16]. During the formation of pulmonary fibrosis, myofibroblasts produce collagens, such as collagen 1A1 and collagen 3A1, as well as major substances that make up the extracellular matrix, such as fibronectin. The overproduction of collagen and extracellular matrix interferes with the normal physiological repair of lung tissue [17].

We hypothesized that CD-NPs have greater acute lung toxicity (inflammation, mitochondrial damage, cell death, and fibrosis) and fibrosis-inducing effects via EMT than do CD-MPs. In this study, the differences between CD-NPs and CD-MPs were used to explore the physicochemical properties affecting the toxicity of CD-NPs. A549 and BEAS-2B cells were used to determine the differences in acute pulmonary toxicity of CD-NPs and CD-MPs in vitro. In vitro and in vivo fibrosis models were constructed to evaluate the difference in the progression of pulmonary fibrosis induced by CD-NPs and CD-MPs and to explore the expression of EMT and pro-fibrotic marker molecules.

## Materials and methods

### Cell lines and culture

Human BEAS-2B cells were purchased from Guangzhou Saiku Biotechnology Co., Ltd., China, and cultured in BEGM complete medium (#CC-3171, LONZA, Switzerland). Human A549 cells were obtained from the American Type Culture Collection (ATCC, Rockville, MD, USA) and cultured in RPMI 1640 medium (Jiangsu Kage Biotechnology Co., Ltd., China) with 10% GIBCO fetal bovine serum (Inüitrogen, USA). Both cell lines were maintained in a 5% CO_2_ incubator at 37 °C.

### Nano-to-micron sized respirable coal dust collection and preparation

The coal samples examined were collected from the Xinji No. 2 mine in China located at longitude 116° 33′ 52ʺ ~ 116° 38′ 17ʺ east and latitude 32° 41′ 04ʺ ~ 32° 43′ 52ʺ north. The mine produces about 2.4 million tons of coal per year and about 440,000 tons of fly ash per year. Samples from each coal face in the ventilated area were thoroughly mixed to obtain composite samples representative of the test area and stored at 4 °C for subsequent analysis. Fresh coal samples were sealed in airtight plastic bags at the mine site and placed in distilled water upon arrival at our laboratory to prevent oxidation. To prepare CD-NPs and CD-MPs samples, the pulverized coal aqueous solution was filtered through UV-sterilized 18,000-mesh (Pore size: 500 nm, Lvbang, Guangdong, China) and 2500-mesh (Pore size: 5 µm, Lvbang, Guangdong, China) filters. Samples filtered through an 18,000-mesh filter were classified as CD-NPs, and samples filtered through a 2500-mesh filter were classified as CD-MPs.

### Detection of physicochemical properties of coal dust

The size and morphology of CD particles were observed using a super-resolution field emission scanning electron microscope (SEM) (Regulus8100, Hitachi, Japan) with a resolution of 1.1 nm/1 kV. The size distribution and Zeta potential of the CD particles were measured with a Malvern Nanoparticle Size Potentiometer (Zetasizer Nano ZS90, UK). The elemental composition of CD particles was analyzed with energy X-ray spectrometer (EDAX-GENESIS, USA) at 20 keV with an analysis area of 1.56 µm^2^. Electron spin resonance (ESR)/elect on paramagnetic resonance (EPR) (JEOL JES FA200, Japan) was used to investigate the changes of oxygen radicals in nano- to micro-scale CD particles. Finally, the changes of functional groups in nano- to micro-scale CD particles were analyzed by infrared spectrometer (Nicolet iS5, ThermoFisher, USA).

### Animals

C57BL/6 male mice, 6–8-week-old [license number SCXK (Yu) 2020-0005], were purchased from Henan Skebes Biotechnology Co., Ltd. and housed in a specific pathogen-free room with ambient temperature controlled (22 ± 2 ℃) environment and circulating light conditions. The animals were allowed free access to food and water. All animal experiments followed the ARRIVE Guidelines and the National Institutes of Health Guidelines for the Care and Use of Laboratory Animals (NIH Publication No. 8023, revised in 1978) and were approved by the Animal Experimentation Ethics Review Committee of Anhui University of Science and Technology (No. 20191013-008). Thirty mice with body weights of 19 ± 1.0 g were randomly divided into four groups after 1 week of adaptive feeding (21 ± 0.8 g). A control group was treated with intranasal instillation of 40 μL phosphate-buffered saline daily. A CD-NPs group was treated with intranasal instillation with 40 μL of CD-NPs suspension (32 μg CD-NPs daily); a CD-MPs group was treated with intranasal instillation with 40 μL of CD-MPs suspension (32 μg CD-MPs daily) for 2 weeks (n = 3), 8 weeks (n = 3) and 12 weeks (n = 3); and a mixed CD group was treated with intranasal instillation with 40 μL mixed CD suspension (32 μg mixed CD daily) for 2 weeks (n = 3). The animals were lightly anesthetized with isoflurane. Unilateral lung tissue was collected and fixed with 2.5% glutaraldehyde overnight at 4 °C, followed by transmission electron microscope (TEM, JEM-1400, JEOL, Japan) to detect cell endocytosis and mitochondrial damage (provided by Hefei Xinle Biotechnology Co., Ltd.). Unilateral lung tissue was fixed with 4% paraformaldehyde at 4 °C overnight and stained with hematoxylin–eosin, Masson, and immunohistochemistry (provided by Hefei Xinle Biotechnology Co., Ltd.). The mice necks and chests were dissected, and one lung lobe was ligated. The trachea was exposed for intubation and washed three times with 1 mL of PBS pre-cooled at 4 °C. The supernatant of bronchoalveolar lavage fluid (BALF) (unilateral lung) and serum were collected for measuring IL-6, IL-1β, TNFα, CXCL2 and TGFβ1 with murine ELISA kits (ABclonal Technology Co., Ltd., Wuhan, China) according to the manufacturer’s instructions.

### Transmission electron microscope and optical microscope detection in vitro

In vitro, the cells were treated with 300 µg/mL mixed CD for 24 h, the old medium was discarded, and the cells were washed three times with PBS. After adding fresh medium, the endocytosis, morphology of cells, and the state of CD adsorbed cells were observed under a microscope. Cell pellets were collected to detect intracellular endocytosis and mitochondrial damage by TEM.

### CCK-8 assay

For cell proliferation inhibition assays, 1 × 10^5^ cells were inoculated into each well of a 96-well plate and cultured at 37 °C in a 5% CO_2_ incubator for 24 h. After the cells were stimulated for 0, 24, 48, 72 h by adding 0, 9.375, 37.5, 75, 150, 300, or 600 µg/mL of CD-NPs or CD-MPs, the cell supernatant was discarded, and fresh complete medium was added. For cell proliferation assays, 1 × 10^3^ cells were inoculated into each well of a 96-well plate and cultured at 37 °C in a 5% CO_2_ incubator for 1, 2, 3, 4, and 5 days. Then, 10 µL of CCK-8 solution (Beyotime, Nanjing, China) was added and cultured for 3 h. The absorbance was measured at 450 nm with a microplate reader.

### ELISA assay in vitro

Cell culture supernatants were collected within 1 h after cells were treated with 300 µg/mL of CD-NPs and CD-MPs for 72 h. Human IL-6, IL-1β, TNFα, CXCL2, and TGFβ1 ELISA kits (ABclonal Technology Co., Ltd., Wuhan China) were used for quantitative detection of cytokines, according to the manufacturer’s protocol.

### JC-1staining, calcium imaging, reactive oxygen species detection, AO/EB staining, and flow cytometry with Annexin V-FITC/PI staining

5 × 10^5^ cells were inoculated into each well of a 24-well plate and cultured at 37 °C in a 5% CO_2_ incubator for 24 h. For JC-1staining, cells were treated with 300 µg/mL of CD-NPs and CD-MPs for 24 h. For AO/EB staining and flow cytometry with Annexin V-FITC/PI staining, cells were treated with 300 µg/mL of CD-NPs and CD-MPs for 72 h. JC-1 stock solution (KeyGEN BioTECH Corp., Ltd., Jiangsu China) was prepared into a working solution, with a final concentration of 8 µg/mL in cell culture medium. The AO/EB working solution was prepared by adding equal volumes of AO (Solarbio, Beijing, China) and EB (KeyGEN BioTECH Corp., Ltd., Jiangsu China) to the cell culture medium at a ratio of 1:19. Annexin V-FITC/PI working solution was prepared by adding 12 µL Annexin V-FITC stock solution (Beyotime, Nanjing, China) and 4 µL propidium iodide stock solution (Beyotime, Nanjing, China) to 200 µL buffer solution. For calcium imaging and detection of reactive oxygen species (ROS), cells were treated with 300 µg/mL of CD-NPs and CD-MPs for 8 h and 12 h before adding their working solutions. Fluo 3-AM stock solution (Dojindo Laboratories, Japan) was prepared into a working solution with a final concentration of 5 µmol/L with Hank’s Balanced Salt Solution. Fluorescent probe DCFH-DA stock solution (Beyotime, Nanjing, China) was added to serum-free cell culture medium at a ratio of 1:1000 to prepare a working solution for the detection of ROS. Described methods were used for subsequent steps [18–22].

### Western blot

The cells were inoculated into 30 cm^2^ dishes at a density of 1 × 10^7^ cells/dish at 37 °C in a 5% CO_2_ incubator for 24 h. After adding various concentrations of CD to stimulate cells for preset times according to grouping requirements, radio immunoprecipitation assay lysis buffer supplemented with protease phosphatase inhibitor cocktail (Beyotime, Nanjing, China) at a ratio of 1:50 was added, and cell proteins were collected. Protein concentration was measured with a BCA kit (Biosharp, Hefei, Anhui, China) according to the manufacturer’s instructions. The protein molecules were separated by electrophoresis on SDS-polyacrylamide gel and transferred to PVDF membrane (Millipore, USA). Membranes were blocked with 5% skim milk for 1 h at room temperature and incubated with primary antibodies HO-1 (1:1000, CST, #5853, USA), β-actin (1:1000, CST, #4970, USA), Caspase 1 (1:1000, CST, #2225, USA), Cleaved Caspase 1 (1:1000, CST, #4199, USA), Bcl-2 (1:1000, CST, #9942, USA), α-SMA (1:1000, CST, #19245, USA), COL-1 (1:2000, Abcam, ab260043, UK), COL-3 (1:1000, Abcam, ab184993, UK), ZO-1 (1:1000, CST, #49398, USA), ZEB1 (1:1000, CST, #49398, USA), N-cadherin (1:1000, CST, #49398, USA), E-cadherin (1:1000, CST, #49398, USA), Vimentin (1:1000, CST, #49398, USA), Slug (1:1000, CST, #49398, USA) and TGFβ1 (1:1000, abclonal, #A2124, China) at 4 °C overnight. The primary antibodies were recovered, and the membrane was washed three times with tris buffered saline and tween and incubated with the secondary antibody for 1 h at room temperature. A luminescence solution (ECL luminescence kit; EMD Millipore) was used for development.

### Cell migration and invasion assays

Scratch healing assay: 8 × 10^5^ cells were inoculated into each well of a 6-well plate with a horizontal line at the bottom for 24 h at 37 °C in a 5% CO_2_ incubator. After scratching with a 10 µL pipette tip perpendicular to the horizontal line at the bottom of the bottle, the old medium was replaced with serum-free medium, and culture was continued for the preset time. Observation and photographing were performed under an inverted microscope (Leica, Germany).

Transwell assay: When the cells had grown to cover 80% of the bottom area of the culture flask, they were starved in serum-free medium for 24 h. The cells were trypsinized to prepare a serum-free cell suspension at a density of 1 × 10^6^ cells/mL, and 100 µL of cell suspension were added to the upper chamber. Then, 600 µL of complete culture medium containing 10% serum were added to the lower chamber for 48 h at 37 °C in a 5% CO_2_ incubator. The medium was discarded, and the cells were washed 3 times with PBS, fixed with 4% paraformaldehyde for 10 min, and stained with 0.1% crystal violet for 10 min. Cell counting and photographing were performed under an inverted microscope (Leica, Germany).

### Indirect immunofluorescence

4 × 10^5^ cells were inoculated into each well of a 24-well plate at 37 °C in a 5% CO_2_ incubator for 24 h. After discarding the cell supernatant, the cells were washed 3 times with PBS and fixed with 4% paraformaldehyde for 30 min. Primary antibody α-SMA (1:500, CST, #19425, USA) was added and incubated overnight at 4 °C. Alexa Fluor 488-conjugated secondary antibody (1:1000, CST, #4412, USA) was added and incubated at room temperature for 1 h in the dark. Cells were examined with an inverted fluorescence microscope (Leica, Germany) with excitation wavelengths of 480–500 nm and emission wavelengths of 525–530 nm.

### Statistical analysis

All experiments were performed in three independent operations with three replicates each. Data are presented as mean ± SD. Statistical differences between the means of two groups were compared by Student’s t-test, and statistical differences among multiple groups were compared by one-way analysis of variance (ANOVA). P < 0.05 was considered statistically significant (*P < 0.05, **P < 0.01, ***P < 0.001). Statistical analysis of experimental data was performed with GraphPad Prism 6 software (GraphPad Software, USA).

## Results

### Chemical and physical properties of the measured coal samples

**Fig. 1:**
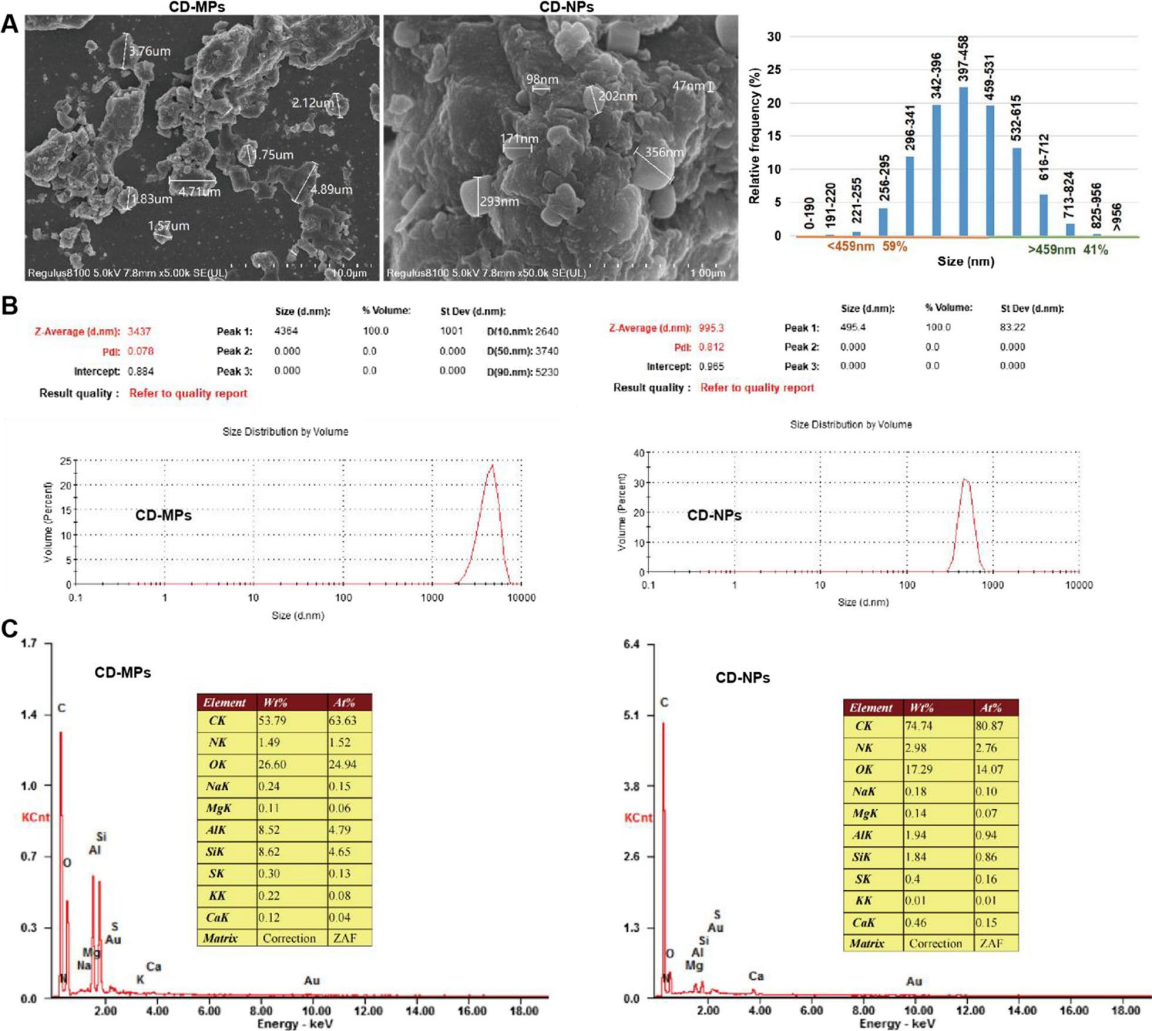
Chemical and physical properties of the measured coal samples. **A** The size and morphology of CD-MPs and CD-NPs were observed with SEM. **B** The size distribution of CD-MPs and CD-NPs were measured with a Malvern Nanoparticle Size Potentiometer. **C** The elemental composition of CD-MPs and CD-NPs was analyzed by energy X-ray spectrometer. *CD-MPs* coal dust micron particles, *CD-NPs* coal dust nanoparticles, *SEM* scanning electron microscopy

Morphological analysis of CD-MPs and CD-NPs by SEM revealed that CD-MPs were irregular, whereas CD-NPs tended to be regular spherical particles (Fig. 1A). The particle size distribution revealed that, by volume, 85.9% of the CD-MPs were smaller than 5 µm, and about 58.9% of the CD-NPs were smaller than 500 nm (Fig. 1B and Additional file 1: Table S1). The EDX spectra of CD-MPs and CD-NPs revealed the presence of several major elemental components such as C, N, O, Al, Si, and other trace elements such as Na, Mg, S, K, Ca, in which the content of silica was less than 10%. However, compared with CD-MPs, the C content in CD-NPs was substantially more, whereas the content of O, Al, Si was less (Fig. 1C). The Zeta potential (Additional file 1: Fig. S1A) and content of oxygen radicals (Additional file 1: Fig. S1B) and functional groups (Additional file 1: Fig. S1C) were not significantly different between of CD-MPs and CD-NPs.

### CD-NPs were more easily endocytosed into cells and inhibited cell proliferation

**Fig. 2:**
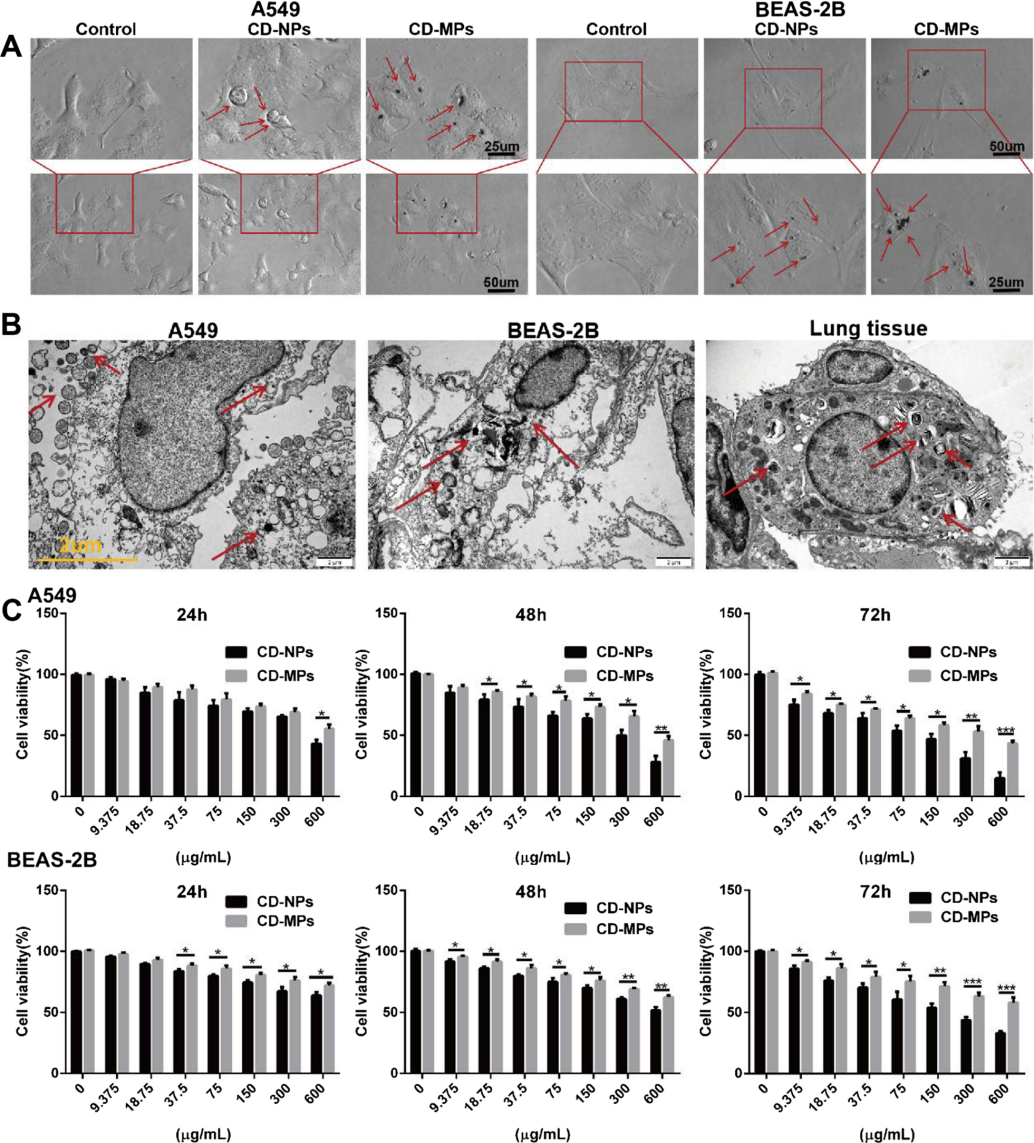
CD-NPs were more easily endocytosed into cells and inhibited cell proliferation. **A** The state of CD adsorbed cells was observed under a microscope after treating with 300 µg/mL CD-MPs and CD-NPs for 24 h. Red arrows point to CD particles adhering to cells. **B** The endocytosis, morphology of cells was observed by TEM after treating with 300 µg/mL mixed CD-MPs and CD-NPs for 24 h in vitro and with 32 μg mixed CD/day for 2 weeks in vivo. Red arrows point to endocytosed CD particles. **C** The cell proliferation was detected by CCK-8 assay. Data were expressed as the mean ± SD, n = 3. *P < 0.05, **P < 0.01 and ***P < 0.001. *CD-MPs* coal dust micron particles, *CD-NPs* coal dust nanoparticles, *CD* coal dust, *TEM* transmission electron microscope

Using light microscopy and TEM, we analyzed the state of CD particles entering cells from a macroscopic and microscopic perspective. The endocytosed CD particles were mostly smaller than 1 μm, indicating that CD-NPs were easily endocytosed into cells (Fig. 2A and B). Next, we used CCK-8 assay to analyze the effects of CD-MPs and CD-NPs on cell proliferation. As illustrated in Fig. 2C, compared with CD-MPs, CD-NPs inhibited the proliferation of alveolar epithelial cells (A549 and BEAS-2B) in a concentration- and time-gradient-dependent manner, indicating that CD-NPs may have a stronger acute pulmonary toxicity.

### CD-NPs released more pro-inflammatory cytokines in vitro and more acute inflammatory response in vivo

**Fig. 3:**
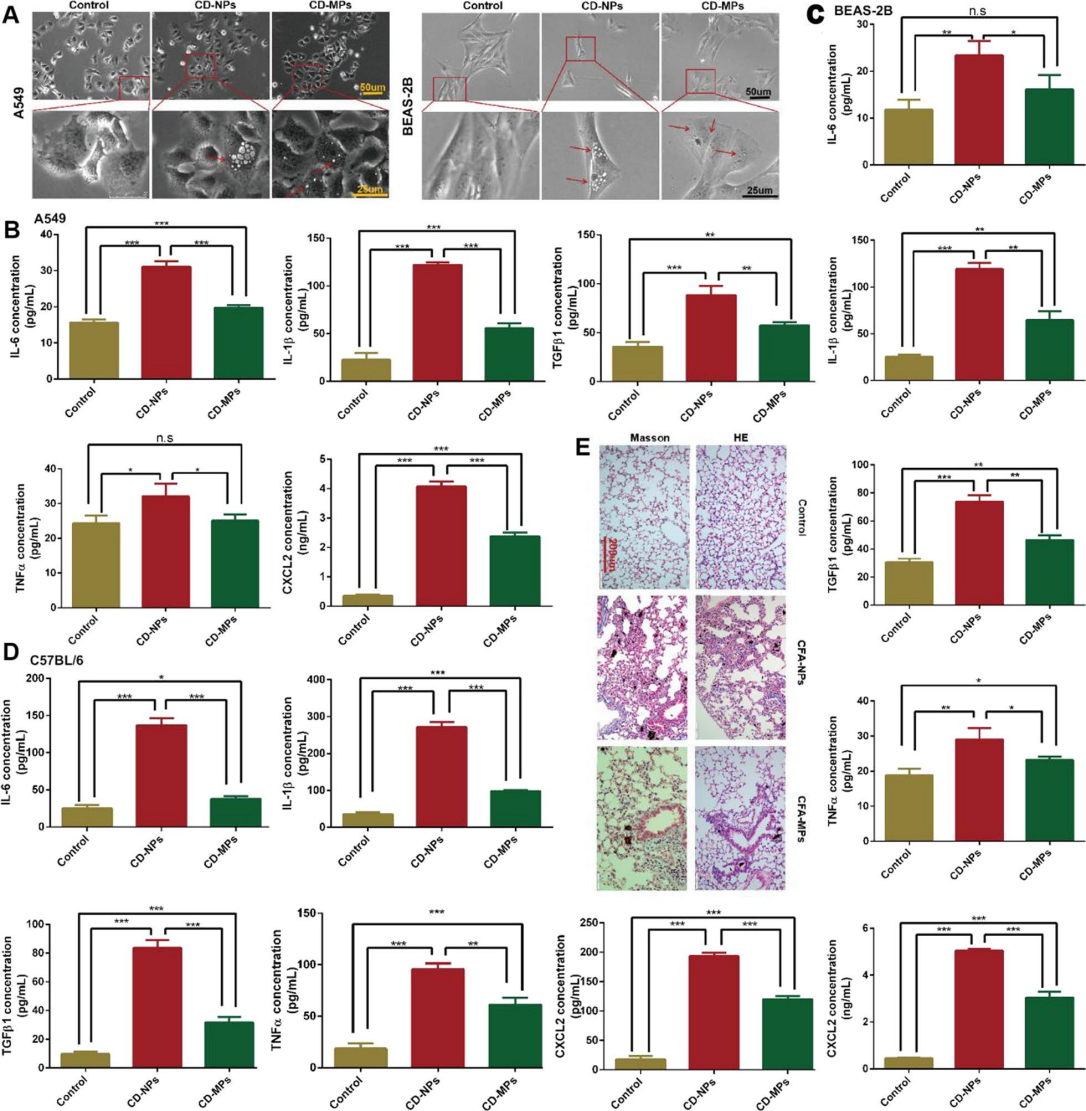
CD-NPs produced a more pronounced acute inflammatory response in vitro and in vivo. **A** The morphology of CD adsorbed cells was observed under a microscope after treating with 300 µg/mL CD-MPs and CD-NPs for 24 h. Red arrows point to vacuoles produced by cells stimulated by CD particles. **B** and **C** Cytokines were quantified in cell supernatants by ELISA assay after treatment with 300 µg/mL CD-MPs and CD-NPs for 72 h in vitro. Data were expressed as the mean ± SD, n = 3. *P < 0.05, **P < 0.01 and ***P < 0.001. *n.s* no significance. **D** Cytokines were quantified in mouse bronchoalveolar lavage fluid by ELISA assay after treatment with 32 μg mixed CD/day for 2 weeks in vivo. Data were expressed as the mean ± SD, n = 3. *P < 0.05, **P < 0.01 and ***P < 0.001. **E** The acute inflammatory hyperplasia in lung tissue was detected by HE and Masson staining after treatment with 32 μg mixed CD/day for 2 weeks in vivo. *CD-MPs* coal dust micron particles, *CD-NPs* coal dust nanoparticles, *CD* coal dust

Through light microscopy, we observed that CD particles, especially CD-NPs, induced vacuolation in the alveolar epithelium (Fig. 3A). After cells were treated with CD-NPs and CD-MPs for 72 h and CD-NPs and CD-MPs were administered intranasally to C57BL/6 mice for 2 weeks, we detected the levels of inflammatory factors IL-6, IL-1β, TNFα, TGFβ1 and chemokine CXCL2 in mouse BALF and the culture supernatant of A549 and BEAS-2B cells with ELISA kit. As shown in Fig. 3B–D, CD particles, especially CD-NPs, induced the release of cellular inflammatory factors and increased the levels of inflammatory factors in BALF. We analyzed whether CD induced acute inflammatory hyperplasia in lung tissue by hematoxylin–eosin and Masson staining: CD stimulation induced acute lung tissue inflammatory hyperplasia, and CD-NPs induced more acute lung tissue inflammatory hyperplasia than did CD-MPs (Fig. 3E). These results demonstrated that CD-NPs produced a more pronounced acute inflammatory response in vitro and in vivo than did CD-MPs.

### CD-NPs more clearly induced acute mitochondrial damage in vitro and in vivo

**Fig. 4:**
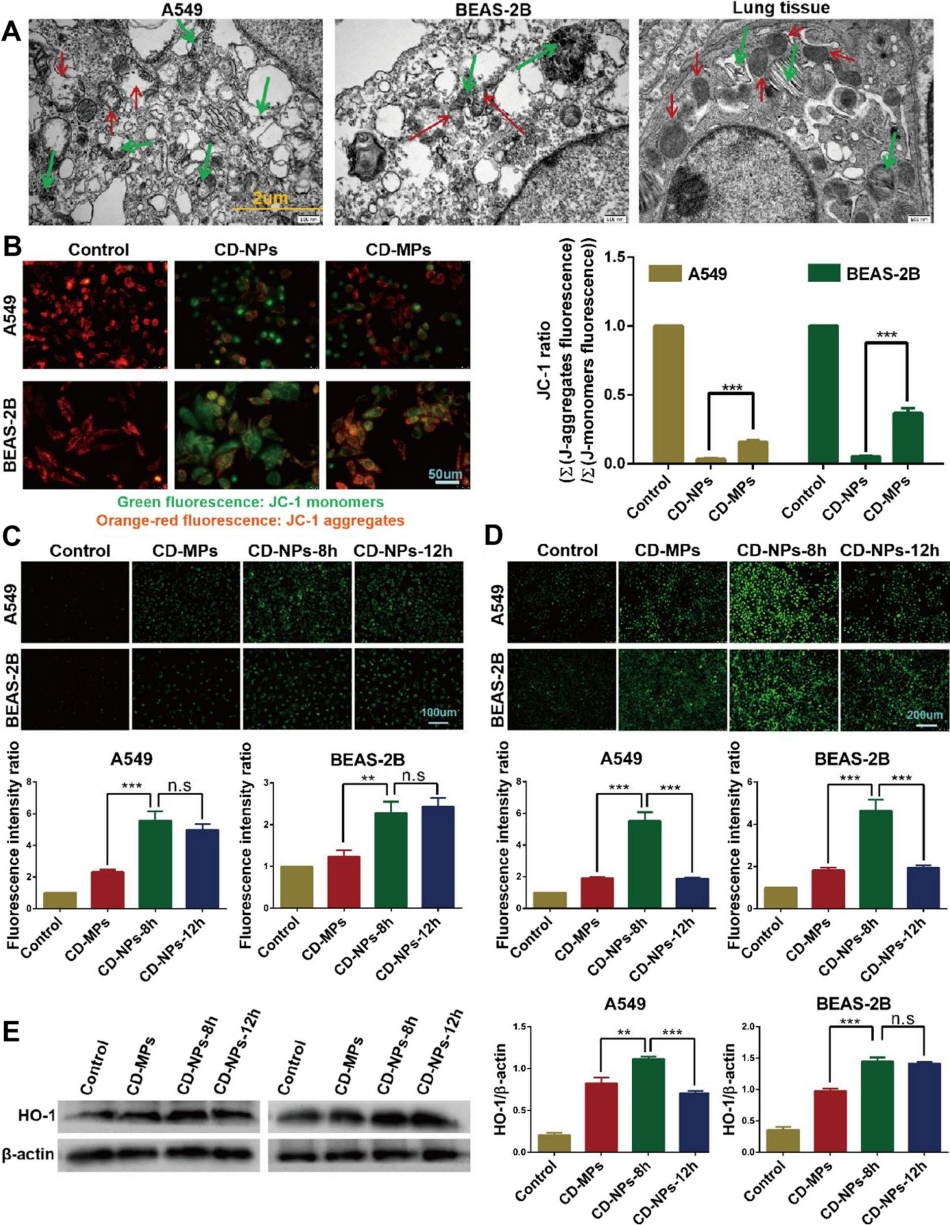
CD-NPs induced acute mitochondrial damage in vitro and in vivo. **A** The mitochondrial damage illustrated by TEM after treatment with 300 µg/mL mixed CD-MPs and CD-NPs for 24 h in vitro and with 32 μg mixed CD/day for 2 weeks in vivo. Green arrows point to endocytosed CD particles. Red arrows point to damaged mitochondria stimulated by CD particles. **B** Mitochondrial membrane potential was detected by JC-1 staining after treatment with 300 µg/mL mixed CD-MPs and CD-NPs for 24 h. Data were expressed as the mean ± SD, n = 3. ***P < 0.001. **C** The content of intracellular free Ca^2+^ was detected by indirect immunofluorescence after treatment with 300 µg/mL mixed CD-MPs for 8 h and 300 µg/mL mixed CD-NPs for 8 and 12 h. Data are expressed as the mean ± SD, n = 3. **P < 0.01 and ***P < 0.001. n.s., no significance. **D** The ROS level of cells was detected by indirect immunofluorescence after treatment with 300 µg/mL mixed CD-MPs for 8 h or 300 µg/mL mixed CD-NPs for 8 and 12 h. Data were expressed as the mean ± SD, n = 3. ***P < 0.001. **E** The level of HO-1 protein was detected by western blot after treatment with 300 µg/mL mixed CD-MPs for 8 h and 300 µg/mL mixed CD-NPs for 8 and 12 h. Data were expressed as the mean ± SD, n = 3. **P < 0.01 and ***P < 0.001. *n.s.* no significance, *CD-MPs* coal dust micron particles, *CD-NPs* coal dust nanoparticles, *CD* coal dust, *TEM* transmission electron microscope, *ROS* reactive oxygen species. Remarks: Due to insufficient experimental funds, in the western blot assay, we cut the PVDF membrane into a membrane small enough so the antibody could be incubated according to its molecular weight and the protein molecular weight marker

TEM observation results showed that after treatment with 300 µg/mL mixed CD for 24 h, mitochondrial membranes were ruptured in alveolar epithelial cells in vitro. Some mitochondria swelled significantly, the interior had inhomogeneous changes, and the orderly structure of cristae was destroyed. In alveolar epithelial cells in vivo, there was matrix-type mitochondrial swelling; that is, the mitochondria were enlarged and rounded, the matrix became lighter, the matrix particles disappeared, the cristae were shortened, reduced, or even disappeared, and the boundary of the mitochondrial membrane was blurred (Fig. 4A). These findings suggest that CD can induce mitochondrial damage in cells during the acute response phase. As shown in Fig. 4A, the CD particles entering the cells were smaller than 1 μm, and most were smaller than 500 nm, which is consistent with the results in Fig. 2B. When active mitochondria are damaged, changes in mitochondrial membrane potential are often accompanied. Therefore, we further examined the change of membrane potential by JC-1 staining. As shown in Fig. 4B, after the cells were stimulated with 300 µg/mL CD-NPs and CD-MPs for 24 h, the mitochondrial membrane potential changed from high to low. (At high potential, JC-1 dye monomers form aggregates that emit red fluorescence at a wavelength of approximately 590 nm and accumulate in mitochondria; at low potential, the JC-1 dye monomer emits green fluorescence at a wavelength of approximately 529 nm).

The results of calcium ion fluorescent probe FLUO-2/AM to measure the content of free calcium ions showed that short-term stimulation of CD particles induced an increase in the content of free calcium ions in cells, especially in CD-NPs, and peaked at 8 h (Fig. 4C). Similarly, the results of intracellular ROS assay showed that short-term stimulation of CD, especially CD-NPs particles, induced an increase in intracellular ROS content, which also reached a peak at 8 h (Fig. 4D). In addition, the protein expression level of heme oxygenase-1 (HO-1) showed a changing trend consistent with the intracellular ROS content (Fig. 4E). Taken together, these results indicate that CD-NPs induced acute mitochondrial damage in vitro and in vivo.

### CD-NPs induced pyroptosis or necrosis in higher proportions in vitro

**Fig. 5:**
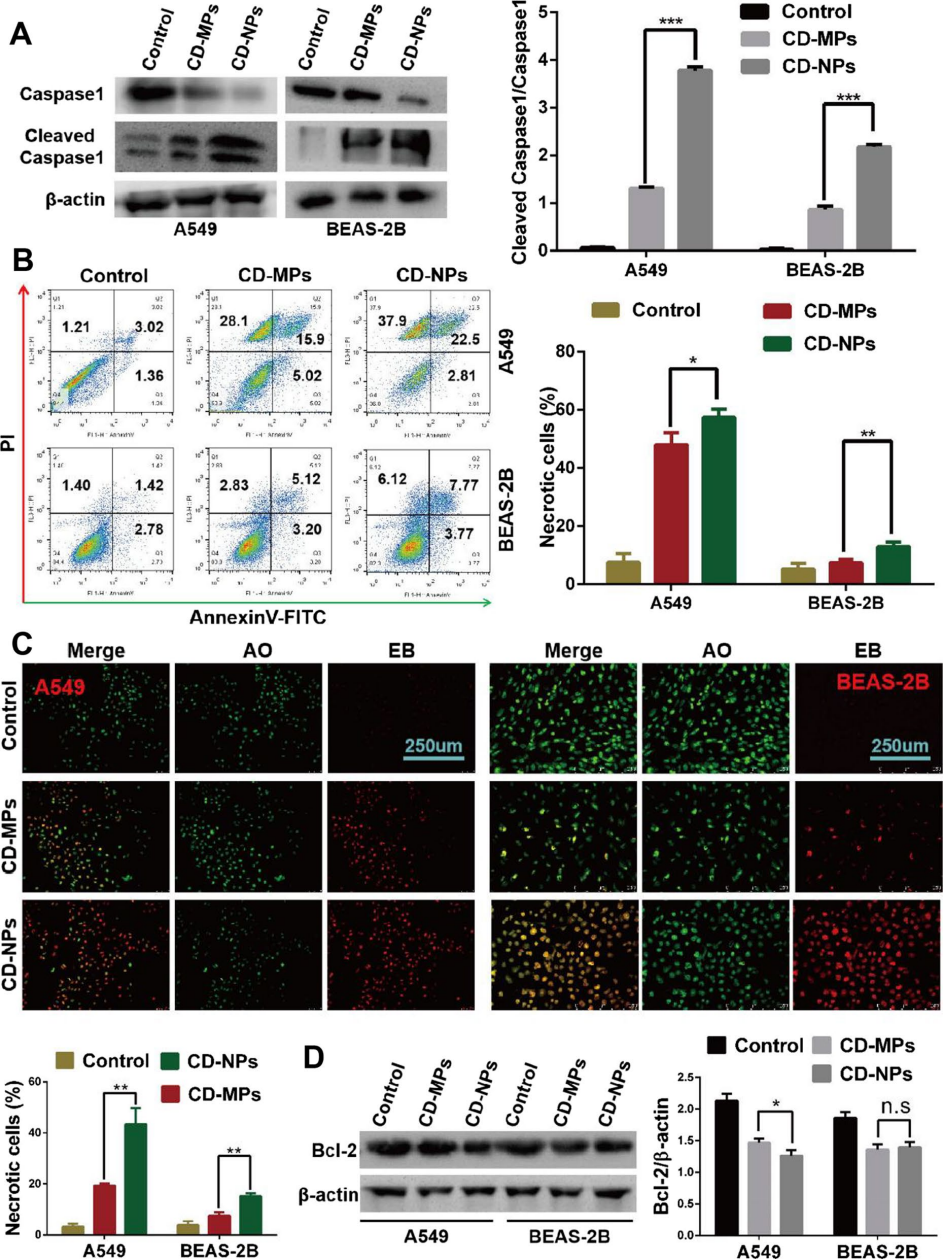
CD-NPs induced pyroptosis or necrosis in higher proportions than CD-MPs in vitro. **A** and **D** The levels of caspase1, cleaved caspase1 and Bcl-2 were examined with western blot after treatment with 300 µg/mL CD-MPs or CD-NPs for 72 h. Data are expressed as the mean ± SD, n = 3. *P < 0.05 and ***P < 0.001. n.s., no significance. Remarks: Due to insufficient experimental funds, in the western blot assay, we cut the PVDF membrane into a membrane small enough to permit incubation of the antibody according its molecular weight and the protein molecular weight marker. **B** and **C** Cell necrosis rate was assessed by flow cytometry with annexin V-FITC/PI staining and AO/EB staining after treatment with 300 µg/mL mixed CD-MPs or CD-NPs for 72 h. Data are expressed as the mean ± SD, n = 3. *P < 0.05 and **P < 0.01. *CD-MPs* coal dust micron particles, *CD-NPs* coal dust nanoparticles

When cells are acutely stimulated by external particles, damage responses such as apoptosis, pyroptosis, autophagy and necrosis occur. In multicellular organisms, cell death is a key process in development, homeostasis, and immune regulation, and its dysregulation is associated with a variety of pathologies. As CD-NPs stimulated alveolar epithelial cells to produce acute inflammatory response, we further examined pro-inflammatory programmed cell death (pyroptosis), accidental cell death (cell necrosis), and non-pro-inflammatory programmed cell death (cell necrosis) in vitro. After cells were treated with 300 µg/mL CD-NPs and CD-MPs for 72 h, the protein expression level of cleaved caspase1/caspase1 was significantly increased, especially in CD-NPs-stimulated cells, a finding indicating that CD-NPs-stimulated cells occurred to a higher degree of caspase-1-dependent pyroptosis (Fig. 5A). Subsequently, annexin V-FITC/PI (Fig. 5B) and AO/EB (Fig. 5C) staining showed that CD stimulation could induce apoptosis and necrosis, and CD-NPs more effectively induced cell necrosis than did CD-MPs. However, neither CD-MPs nor CD-NPs induced early apoptosis. Finally, we examined the expression level of the anti-apoptotic protein Bcl-2. Although Bcl-2 inhibition occurred in cells briefly stimulated with CD particles (300 µg/mL CD-NPs and CD-MPs for 72 h) compared with untreated cells, Bcl-2 expression levels were only slightly lower in CD-MPs-stimulated A549 cells (P < 0.05), while there was no significant difference (P > 0.05) in BEAS-2B cells (Fig. 5D). These results indicated that short-term CD-NPs stimulation induced pyroptosis and necrosis, which may be the reason for the detection of higher amounts of inflammatory factors in the supernatant of short-term CD-NPs-stimulated cells.

### CD-NPs induced EMT and pro-fibrogenesis in vitro

**Fig. 6:**
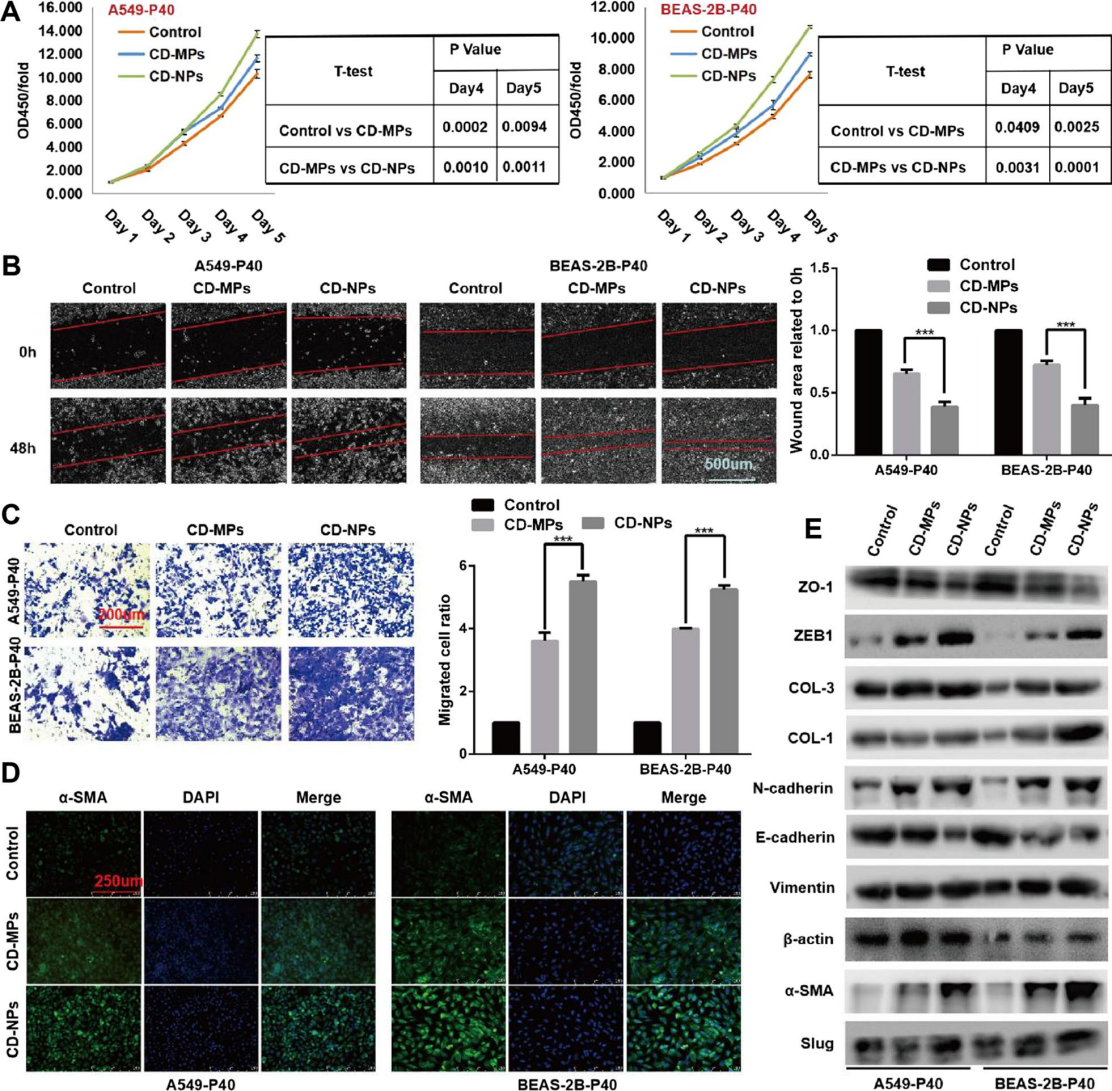
CD-NPs induced EMT and pro-fibrogenesis in vitro. **A** Cell proliferation was detected with CCK-8 assay. Data were expressed as the mean ± SD, n = 3. **B** Cell migration was detected by scratch healing assay. Data were expressed as the mean ± SD, n = 3. ***P < 0.001. **C** Cell invasion was detected with Transwell assay. Data ware expressed as the mean ± SD, n = 3. ***P < 0.001. **D** α-SMA were detected with indirect immunofluorescence. **E** EMT and pro-fibrogenesis marker molecules was detected with western blot. Remarks: Due to insufficient experimental funds, in the western blot assay, we cut the PVDF membrane into a membrane small enough to permit incubation of the antibody according to its molecular weight and the protein molecular weight marker. *CD-MPs* coal dust micron particles, *CD-NP* coal dust nanoparticles, *EMT* epithelial–mesenchymal transition, *P0* passage 0 cells, *P10* passage 10 cells, *P20* passage 20 cells, *P40* passage 40 cells

The cells were stimulated with 9.375 µg/mL of CD-MPs or CD-NPs for 3 consecutive days, then passaged. The 0 passages (P0), 10 passages (P10), 20 passages (P20), and 40 passages (P40) cells with different fibrotic states were induced by CD-NPs according to above cycle. Compared with P0 cells, the migration viability of P10, P20, and P40 cells, especially P40 cells, induced by CD-NPs was increased (Additional file 1: Fig. S2A). Similarly, the proliferative (Additional file 1: Fig. S2B) and invasive (Additional file 1: Fig. S2C) abilities of P10, P20, and P40 cells, especially of P40 cells, were enhanced compared to those qualities of P0 cells induced by CD-NPs. In addition, compared with alveolar epithelial cells without CD-NPs induction, EMT marker molecules (E-cadherin and ZO-1) were significantly down-regulated in CD-NPs-induced P40 alveolar epithelial cells, whereas EMT marker molecules (N-cadherin, ZEB1, vimentin, and Slug) and pro-fibrogenesis marker molecules (COL-1, COL-3 and α-SMA) were significantly upregulated (Additional file 1: Fig. S2D). The above results indicated that compared to P0, P10 and P20 cells, the EMT and pro-fibrogenesis states of P40 cells were the most significant. Therefore, we chose P40 cells as an in vitro model. Next, we measured the pre-fibrotic states of P40 cells induced by CD-MPs and CD-NPs to analyze the difference in the ability of CD-MPs and CD-NPs to induce EMT and pre-fibrosis in alveolar epithelial cells. The results showed that compared with CD-MPs-induced P40 cells, CD-NPs-induced P40 cells had more proliferation activity (Fig. 6A), more migration (Fig. 6B), and invasion (Fig. 6C) abilities and expression of EMT markers (E-cadherin, N-cadherin, ZO-1, ZEB1, Vimentin and Slug) and pro-fibrogenesis markers (COL-1, COL-3 and α-SMA) (Fig. 6D and E). Thus, CD-NPs are more chronically toxic than are CD-MPs, i.e., promotion of the progression of pulmonary fibrosis, as recorded by EMT, in vitro.

### CD-NPs more distinctly induced EMT and pro-fibrogenesis in vivo

**Fig. 7:**
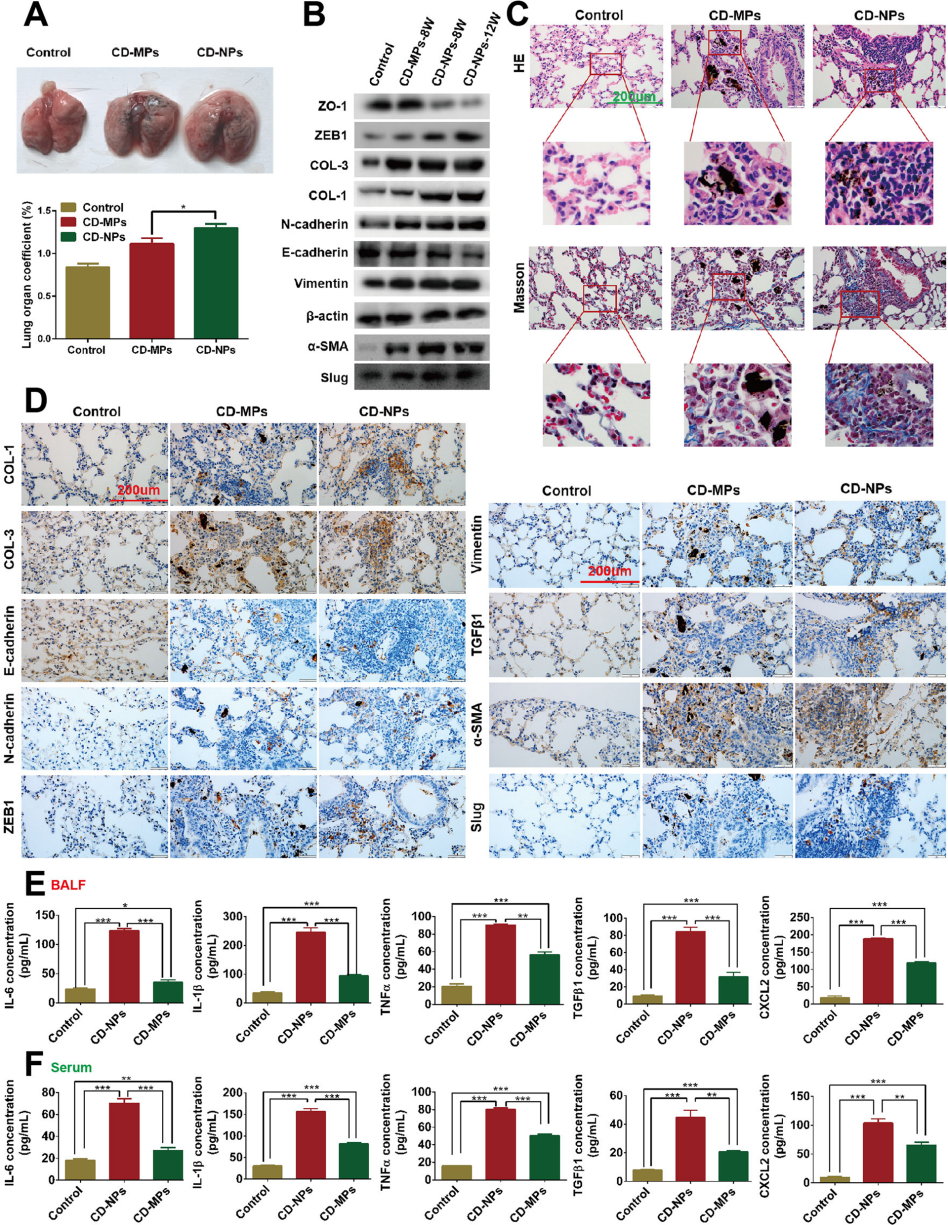
CD-NPs induced EMT and pro-fibrogenesis in vivo. **A** Representative image of lung appearance and lung coefficient in mice after 8 weeks of nasal feeding of CD-MPs or CD-NPs. Data are expressed as the mean ± SD, n = 3. *P < 0.05. **B** The levels of EMT and pro-fibrogenesis marker molecules of mouse lung tissue were detected with western blot after 8 and 12 weeks of nasal feeding of CD-MPs or CD-NPs. Remarks: Due to insufficient experimental funds, in the western blot assay, we cut the PVDF membrane into a membrane small enough to permit incubation of the antibody according to its molecular weight and the protein molecular weight marker. **C** Hematoxylin–eosin and Masson staining were performed after 12 weeks of nasal feeding of CD-MPs or CD-NPs. **D** EMT and pro-fibrogenesis marker molecules of mouse lung tissue were detected by immunohistochemistry after 12 weeks of nasal feeding of CD-MPs or CD-NPs. **E** and **F** Cytokines were quantified in mouse BALF and serum with ELISA assay after 12 weeks of nasal feeding of CD-MPs or CD-NPs. Data are expressed as the mean ± SD, n = 3. *P < 0.05, **P < 0.01 and ***P < 0.001. *CD-MPs* coal dust micron particles, *CD-NPs* coal dust nanoparticles, *EMT* epithelial–mesenchymal transition, *BAL* bronchoalveolar lavage fluid

According to the National Occupational Health Standards of the People’s Republic of China—Occupational Exposure Limits for Hazardous Factors in the Workplace (GBZ2.1-2007), promulgated by the Ministry of Health in 2007, the concentration limit of respirable CD (free SiO_2_ < 10%) is 2.5 mg/m^3^. According to the daily inhalation rate of adults is 16 m^3^, the legal working day of the country is 8 h/day, and the equivalent dose conversion formula between standard animals is Dose (mice) = k × Dose (human), k = 0.0025. We calculated that the daily dose of CD inhaled by mice is Dose (mice) = 0.0025 × (2.5 mg/m^3^ × 16 m^3^ × 8 h/24 h) = 32 µg. Therefore, the dose of 32 µg/40 uL/day of CD was used for nasal feeding mice for 8 and 12 weeks to establish pre-fibrosis in vivo. The mice were sacrificed and weighed. The lung tissue was removed and weighed after the trachea and surrounding connective tissue were stripped. The lung coefficient (%) = lung tissue weight/mice body weight × 100%. As shown in Fig. 7A, the appearance of lung tissues in the control group was pink, and the texture was soft, whereas the lung tissues in the CD-NPs animals had many fine coal spots, and lung tissues in the CD-MPs animals had many sparse and coarse coal spots. Hematoxylin–eosin and Masson staining showed that compared with the control group and the CD-MPs group, the lung tissues of the CD-NPs group had more nodules and bluer collagen (Fig. 7C). To further analyze whether EMT promotes the formation of pre-fibrosis in vivo, we found that compared with the control group, the expression levels of EMT marker molecule (E-cadherin and ZO-1) in the lung tissue of mice induced by CD were less, whereas the expression levels of other EMT markers (N-cadherin, ZEB1, Vimentin and Slug), pro-fibrogenesis markers (COL-1, COL-3 and α-SMA), and EMT-inducing factors (TGFβ1) were more, as recorded by western blot (Fig. 7B) and immunohistochemical staining (Fig. 7D), especially in the CD-NPs animals, which was consistent with the in vitro results. Finally, the results of ELISA showed many inflammatory factors produced in the bronchoalveolar lavage fluid (Fig. 7E) and serum (Fig. 7F) of mice by long-term exposure with CD, especially CD-NPs. Based on the above results, we conclude that long-term stimulation by CD particles, especially by CD-NPs, can promote the formation of pulmonary fibrosis in vivo through the EMT pathway. In other words, CD-NPs are more chronically toxic than CD-MPs, i.e., promotion of the progression of pulmonary fibrosis by EMT in vivo.

## Discussion

An important problem faced in coal mining is the generation of inhalable dust particles. An important quality of coal dust (CD) is particle size, which directly affects its reaction rate, sedimentation, solubility, and, ultimately, human health [23, 24]. Accurate characterization of CD particle size is important in attempts to prevent and control the health hazards of CD pollution.

In this study, CD nanoparticles (CD-NPs) were all less than 1 μm, and about 59% of the particles were less than 500 nm by volume. CD micron particles (CD-MPs) are all larger than 1 µm, of which about 86% were smaller than 5 µm by volume. This particle size is in the range of that of respirable CD, which can be deposited in the lower respiratory tract by gravity and adhere to the alveolar walls by Brownian motion (diffusion) [25]. Coal worker’s pneumoconiosis and progressive massive fibrosis are synergistic effects of coal, silica dust, rock dust particles and diesel particulate matter. The composition of CD particles varies greatly among regions [2]. In this study, we focused on the effect of coal particle size on its toxicity and cellular responses. The particle size distribution revealed that the two kinds of CD particles we collected meet the requirements that the particle size of most CD-NPs is less than 500 nm, while the particle size of most CD-MPs is more than 1 μm and less than 5 μm. The significance of Zeta potential is that its value is related to the stability of colloidal dispersion and is a measure of the strength of mutual repulsion or attraction between particles: the smaller the molecule or dispersed particle, the higher the absolute value of Zeta potential and the more stable the system; that is, the dissolution or dispersion can resist aggregation. The larger the molecules or dispersed particles, the lower the absolute value of Zeta potential and the more prone is coagulation or cohesion; that is, the attractive force exceeds the repulsive force, dispersion is disrupted, and coagulation occurs. The absolute value of the Zeta potential (22.8 mV) of CD-NPs is slightly higher than that of CD-MPs (− 17.5 mV), which is consistent with the principle that the smaller the particle, the higher the absolute value of the Zeta potential. However, the Zeta potentials of both CD-NPs and CD-MPs ranged from ± 10 to ± 30 mV, at which both particles became unstable from rapid coagulation or agglomeration. SEM verified the particle size of CD-NPs and CD-MPs and showed that both CD-NPs and CD-MPs were coagulated or agglomerated; only a few particles were dispersed, which was consistent with the analysis of Zeta potential.

Energy X-ray spectroscopy analysis showed that the content of silica in CD was less than 10%, which meets the requirements of CD composition. The content of C in CD-NPs was significantly more than in CD-MPs, whereas the contents of O, Al, and Si were less, as found in previous research [2]. Combined with the results of previous studies [26, 27], it can be concluded that compared with CD-MPs, the reduced oxygen-to-carbon ratio in CD-NPs indicates that its wettability is lower than that of CD-MPs. Coal workers regularly inhale respirable coal particles due to the lower probability that respirable coal particles with lower wettability will be wetted by the water spray and then dispersed in the water. Furthermore, with decreasing particle size, the particle number increases, and the particle weight decreases, indicating a much higher number of respirable coal particles. Therefore, from a transportation point of view, the toxicity potential of coal with reduced particle size is increased. Contrary to previous studies [2], we found that the functional groups and oxygen radicals of CD-MPs and CD-NPs did not differ significantly. In addition, small particles have a larger surface area. Smaller particles with larger surface area have high capacity to play a key role in nanoparticle-induced lung injury [28, 29]. Characterization analysis of CD in this study indicated that CD-NPs may have higher toxicity due to lower oxygen-to-carbon ratio, smaller size, and larger surface area.

To analyze the toxicity of CD to cells, we first used an optical microscope to detect the state of adsorption of CD-NPs and CD-MPs on cells. The surface of cells treated with CD-NPs had adsorption of fine CD particles, while the surface of cells treated with CD-MPs had adsorption of coarse CD particles, indicating that CD particles can adsorb onto cells. It also reflects the difference in the particle size of the two types of CD. In addition, by TEM, it was found that the cells were able to endocytose CD particles, and the particles entering cells through endocytosis were mostly smaller than 1 µm, indicating that small CD particles are more likely to enter cells. Both CD-NPs and CD-MPs, especially CD-NPs, can inhibit the proliferation of cells in a concentration- and time-dependent manner, which may be related to the easier entry of CD-NPs into cells. When the concentration of CD-NPs and CD-MPs was 9.375 µg/mL, three consecutive days of stimulation had little effect on the proliferation of cells, whereas when the concentration of CD-NPs and CD-MPs was 300 µg/mL, three consecutive days of stimulation significantly inhibited cell proliferation. Therefore, in the follow-up study, we selected 9.375 µg/mL of CD-NPs and CD-MPs to induce cells long-term for construction of a pre-fibrosis model to be used in analysis of the chronic pulmonary toxicity of CD particles. At the same time, we selected 300 µg/mL of CD-NPs and CD-MPs to induce cells for 72 h for analysis of the acute pulmonary toxicity of CD particles and used 300 µg/mL CD-NPs and CD-MPs to induce cells for 24 h to examine cell morphology.

In the process of cell culture, due to insufficient serum concentration, drug action, external stimuli, and other factors affecting cell metabolism, cell vacuolization will occur under stress of the endoplasmic reticulum [30, 31]. The accumulation of unfolded proteins within the endoplasmic reticulum creates stress, and the unfolded protein response stimulates inflammatory pathways to alert surrounding cells of potential danger [32]. The transient interaction between impaired protein homeostasis and inflammation may be a beneficial feature of unfolded protein response [33]. However, persistent UPR-induced inflammation is considered a pathological factor in many chronic diseases [33, 34]. The development and occurrence of inflammatory responses are closely related to endoplasmic reticulum stress [32]. Therefore, to evaluate the difference in acute inflammatory response between CD-NPs and CD-MPs induced cells, we first detected the cell morphology induced by 300 µg/mL of CD-NPs and CD-MPs for 24 h by light microscopy. CD-NPs cells had the largest number and largest volume of vacuoles, suggesting that CD-NPs are more likely than CD-MPs to induce ERS and may induce a greater cellular inflammatory response. Furthermore, we detected the expression of cellular inflammatory factors in alveolar epithelial cell supernatant and mouse BALF. Both CD-NPs and CD-MPs induced the secretion of inflammatory factors in cells and lung tissues, and the effect of CD-NPs was more than that of CD-MDs. Hematoxylin–eosin and Masson staining showed that the mouse lung tissue had inflammatory hyperplasia after 2 weeks of CD-NPs exposure, whereas the tissue had mild inflammatory hyperplasia after 2 weeks of CD-MPs exposure. In conclusion, CD-NPs induce more acute inflammatory response in vitro and in vivo than do CD-MPs.

The entry of dust particles into cells can cause mitochondrial damage [35, 36]. Changes in the number, size and structure of mitochondria often occur when cells are damaged by toxicants. In this study, we found that CD treatment induced significant mitochondrial enlargement, abnormal cristae, and concentric vortex. Damaged features of mitochondria include changes in membrane potential and changes in mitochondrial redox potential. JC-1 staining showed that CD particles induced changes in mitochondrial membrane potential, and the effect of CD-NPs was more pronounced than that of CD-MPs. Mitochondria are important mediators of cellular metabolism and are the producers and targets of ROS. By-products of normal mitochondrial metabolism and homeostasis include the accumulation of potentially damaging levels of ROS, Ca^2+^, and other factors, which can lead to the release of ROS and Ca^2+^ when mitochondria are damaged. HO-1 is considered a major protein in diseases caused by oxidative and inflammatory damage. HO-1 has anti-inflammatory, anti-oxidative, anti-apoptotic, and anti-proliferative effects. As an adaptive and protective response to noxious stimuli, the production of HO-1 is closely related to some diseases [37, 38]. In this study, CD particles induced an increase in cellular ROS, Ca^2+^, and HO-1 levels, and the effect was more pronounced for CD-NPs than for CD-MPs. Thus, alveolar epithelial cells produced oxidative, inflammatory damage, and acute mitochondrial damage when they were stimulated by CD, especially by CD-NPs.

Damage of active mitochondria is the precursor step of cell death. Pyroptosis and necrosis are the death modes that can cause cellular inflammatory response, whereas apoptosis is the cell death mode that does not cause inflammatory response. Thus, is it because CD-NPs induce pyroptosis and necrosis during the acute response phase that causes the massive release of inflammatory factors? The process of pyroptosis is caspase-1 dependent. In this study, CD-NPs cells had higher levels of cleaved caspase-1/caspase-1, indicating that CD-NPs had a stronger ability to induce caspase-1-dependent pyroptosis than did CD-MPs. When caspase-1 is activated, cells release inflammatory factors IL-1β and IL-18, which in turn attract more inflammatory cells and aggravate the inflammatory response. According to the staining principle, we counted the cells with FITC/PI double staining and PI single staining as necrotic cells in annexinV-FITC/PI staining and counted the cells with uniform red staining as necrotic cells in AO/EB staining. Both staining reactions showed that CD particles can induce cell necrosis, but CD-NPs induced more cell necrosis. Compared with the control group, the expression level of the pro-apoptotic protein Bcl-2 was significantly lower in the CD particle-induced group, but the difference between CD-NPs and CD-MPs was small, indicating that CD-NPs may induce more severe inflammatory response mainly by inducing a higher proportion of pyroptosis and necrosis.

After the acute inflammatory reaction, CD particles cannot be completely removed, and the body will develop serious chronic diseases, such as CWP and PMF, under long-term chronic toxicity [39, 40]. In this study, A549 cells were used as an in vitro model of type II alveolar epithelial cells. We first identified CD-NPs-induced alveolar epithelial cells at passage 40 as an in vitro pre-fibrosis model by evaluating the proliferation, migration, and invasion abilities, and expression levels of EMT markers and pro-fibrotic proteins of passages 0, 10, 20, and 40 cells induced by CD-NPs. To compare the differences between CD-NPs and CD-MPs in chronic toxicity in cells, we again examined the proliferation, migration, and invasion abilities of alveolar epithelial cells induced by CD-NPs and CD-MPs in passage 40 as well as the expression levels of EMT and profibrotic protein molecules. CD-NPs were found to have a stronger pro-fibrotic effect than did CD-MPs. We simultaneously detected the expression levels of EMT and profibrotic protein molecules in alveolar epithelial cells at passages 0, 10, 20, and 40 that were not induced by CD-NPs to exclude the effect of cell passage. In A549 cells, the expression levels of ZEB1, N-cadherin, α-SMA, and Slug increased with the passage of cells; in BEAS-2B cells, the expression levels of ZO-1 and E-cadherin decreased with the passage of cells, and the expression levels of Slug increased with the passage of cells. Furthermore, the expression levels of EMT marker molecules and profibrotic protein molecules were significantly different between non-dust-induced and coal-dust-induced cells of the same passage. This finding indicates that alveolar epithelial cells can undergo EMT with the passage of cells but does not refute the conclusion that CD particles more significantly induce EMT and profibrotic in vitro.

Organ coefficient is a commonly used index in toxicology experiments. In the present study, the lung index of mice in the CD particle-induced group was significantly increased, especially for CD-NPs, indicating that CD-NPs have more chronic lung toxicity. The most striking feature of lung fibrosis is the formation of nodules and collagen [41]. As a small number of nodules and blue collagen appeared in the CD-NPs-induced lung tissue by hematoxylin–eosin and Masson staining, we concluded that the in vivo pre-fibrosis model had been constructed. The higher expression levels of EMT and pro-fibrotic marker molecules in CD-NPs than in CD-MPs, indicated that CD-NPs could more significantly affect pro-fibrosis progress in vivo by EMT. In addition, the long-term induction of CD-NPs particles increased more the expression of inflammatory factors in mouse BALF and serum than CD-MPs, but expression was significantly less than in the acute response phase. The above results suggest that chronic inflammation plays a role in CD-NPs-induced pre-fibrosis in mice. However, the relationship of chronic inflammation with EMT and the extent to which both play a role in the development of pulmonary fibrosis remains to be determined. Since bronchoalveolar lavage is ineffective for pneumoconiosis, EMT may predominate in pneumoconiosis progression.

To aid the reader, we have made a table (Additional file 1: Table S2) to compare the readouts for the CD-NPs and CD-MPs (not only with P values, but with estimates of the size of the effects and differences).

This study has shortcomings. No “positive” control particles, e.g., crystalline silica, were used to put the results in perspective. The mechanism of CD-NPs-induced pulmonary fibrosis has not been studied. Also, the effect and mechanism of specific elements in coal dust on acute pulmonary toxicity and pulmonary fibrosis have not been analyzed. We will address these issues in follow-up study.

## Conclusions

In this study, CD-MPs and CD-NPs were size segregated, and their characteristics were analyzed comprehensively. Stimulation with CD-NPs led to more severe acute lung toxicity, including acute inflammatory response, mitochondrial damage, pyroptosis, and necrosis than did CD-MPs. In vitro and in vivo models of CD pre-fibrosis were established and showed, for the first time, that stimulation with CD-NPs can induce pulmonary fibrosis through EMT. The strong toxic effect of CD-NPs may be related to their small size, small oxygen-to-carbon ratio, low wettability, and easy accumulation in vivo. The information on the different effects of CD particles of various sizes on lung toxicity may lead to new ideas in the campaign to prevent the harm of coal dust to human health and in treating pneumoconiosis.

## Supplementary Information

**Additional file 1: Table S1.** Particle size distribution of CD-MPs and CD-NPs by volume. **Fig. S1.** Chemical and physical properties of the measured coal samples. A. The Zeta potential of the CD-MPs and CD-NPs were measured with a Malvern Nanoparticle Size Potentiometer. B. ESR/EPR was used to investigate the oxygen radicals of CD-MPs and CD-NPs. C. The functional groups of CD-MPs and CD-NPs were analyzed by infrared spectrometer. CD-MPs, coal dust micron particles; CD-NPs, coal dust nanoparticles; ESR, electron spin resonance; EPR, electron paramagnetic resonance. **Fig. S2.** CD-NPs-induced passage 40 cells were constructed as an in vitro fibrosis model. A. The cell migration was detected with a scratch healing assay. Data were expressed as the mean ± SD, n = 3. *P < 0.05, **P < 0.01 and ***P < 0.001. B. Cell proliferation was detected with CCK-8 assay. Data were expressed as the mean ± SD, n = 3. C. Cell invasion was detected with Transwell assay. Data were expressed as the mean ± SD, n = 3. *P < 0.05, **P < 0.01 and ***P < 0.001. D. The levels of EMT and pro-fibrogenesis marker molecules were detected by western blot. Remarks: Due to insufficient experimental funds, in the western blot assay, we cut the PVDF membrane into a membrane small enough to permit incubation of the antibody according to its molecular weight and the protein molecular weight marker. CD-MPs, coal dust micron particles; CD-NPs, coal dust nanoparticles; EMT, epithelial–mesenchymal transition; P0, passage 0 cells; P10, passage 10 cells; P20, passage 20 cells; P40, passage 40 cells. **Table S2.** Differences in the characteristics and pulmonary toxicity of nano- and micron-sized respirable coal dust (summary of data and effects).

## Data Availability

The datasets used and/or analysed during the current study are available from the corresponding author on reasonable request.

## References

[1] Zhou H, Bhattarai R, Li Y, Si B, Dong X, Wang T (2022). Towards sustainable coal industry: turning coal bottom ash into wealth. Sci Total Environ.

[2] Zhang R, Liu S, Zheng S (2021). Characterization of nano-to-micron sized respirable coal dust: particle surface alteration and the health impact. J Hazard Mater.

[3] Tallec K, Blard O, González-Fernández C, Brotons G, Berchel M, Soudant P (2019). Surface functionalization determines behavior of nanoplastic solutions in model aquatic environments. Chemosphere.

[4] Lim CH, Kang M, Han JH, Yang JS (2012). Effect of agglomeration on the toxicity of nano-sized carbon black in sprague-dawley rats. Environ Health Toxicol.

[5] Sarver E, Keles C, Rezaee M (2019). Characteristics of respirable dust in eight appalachian coal mines: a dataset including particle size and mineralogy distributions, and metal and trace element mass concentrations. Data Brief.

[6] Keles C, Taborda MJ, Sarver E (2022). Updating, "Characteristics of respirable dust in eight Appalachian coal mines: a dataset including particle size and mineralogy distributions, and metal and trace element mass concentrations" with expanded data to cover a total of 25 US mines. Data Brief.

[7] Wu Q, Han L, Xu M, Zhang H, Ding B, Zhu B (2019). Effects of occupational exposure to dust on chest radiograph, pulmonary function, blood pressure and electrocardiogram among coal miners in an eastern province, China. BMC Public Health.

[8] Lu C, Dasgupta P, Cameron J, Fritschi L, Baade P (2021). A systematic review and meta-analysis on international studies of prevalence, mortality and survival due to coal mine dust lung disease. PLoS ONE.

[9] Zhang X, Zhang Z, Wang P, Xiao S, Han K, Tang Y (2021). Comparison of properties of dust in alveolar of rats and the workplace. Exp Lung Res.

[10] Strieter RM, Mehrad B (2009). New mechanisms of pulmonary fibrosis. Chest.

[11] Meyer KC (2017). Pulmonary fibrosis, part I: epidemiology, pathogenesis, and diagnosis. Expert Rev Respir Med.

[12] Hewlett JC, Kropski JA, Blackwell TS (2018). Idiopathic pulmonary fibrosis: epithelial–mesenchymal interactions and emerging therapeutic targets. Matrix Biol.

[13] Parimon T, Yao C, Stripp BR, Noble PW, Chen P (2020). Alveolar epithelial type II cells as drivers of lung fibrosis in idiopathic pulmonary fibrosis. Int J Mol Sci.

[14] Goldmann T, Zissel G, Watz H, Drömann D, Reck M, Kugler C (2018). Human alveolar epithelial cells type II are capable of TGFβ-dependent epithelial–mesenchymal-transition and collagen-synthesis. Respir Res.

[15] Selman M, Pardo A (2020). The leading role of epithelial cells in the pathogenesis of idiopathic pulmonary fibrosis. Cell Signal.

[16] Winters NI, Burman A, Kropski JA, Blackwell TS (2019). Epithelial injury and dysfunction in the pathogenesis of idiopathic pulmonary fibrosis. Am J Med Sci.

[17] Redente EF, Black BP, Backos DS, Bahadur AN, Humphries SM, Lynch DA (2021). Persistent, progressive pulmonary fibrosis and epithelial remodeling in mice. Am J Respir Cell Mol Biol.

[18] Zhang Y, Xie C, Li A, Liu X, Xing Y, Shen J (2019). PKI-587 enhances chemosensitivity of oxaliplatin in hepatocellular carcinoma through suppressing DNA damage repair pathway (NHEJ and HR) and PI3K/AKT/mTOR pathway. Am J Transl Res.

[19] Zhang YC, Wu CG, Li AM, Liang Y, Ma D, Tang XL (2021). Oxaliplatin and gedatolisib (PKI-587) co-loaded hollow polydopamine nano-shells with simultaneous upstream and downstream action to re-sensitize drugs-resistant hepatocellular carcinoma to chemotherapy. J Biomed Nanotechnol.

[20] Zhang Y, Qian X, Yang X, Niu R, Song S, Zhu F (2020). ASIC1a induces synovial inflammation via the Ca2+/NFATc3/ RANTES pathway. Theranostics.

[21] Cao X, Fu M, Bi R, Zheng X, Fu B, Tian S (2021). Cadmium induced BEAS-2B cells apoptosis and mitochondria damage via MAPK signaling pathway. Chemosphere.

[22] Gao L, Xu Z, Huang Z, Tang Y, Yang D, Huang J (2020). CPI-613 rewires lipid metabolism to enhance pancreatic cancer apoptosis via the AMPK-ACC signaling. J Exp Clin Cancer Res.

[23] Trechera P, Moreno T, Córdoba P, Moreno N, Zhuang X, Li B (2020). Mineralogy, geochemistry and toxicity of size-segregated respirable deposited dust in underground coal mines. J Hazard Mater.

[24] Liu T, Liu S (2020). The impacts of coal dust on miners' health: a review. Environ Res.

[25] Darquenne C (2020). Deposition mechanisms. J Aerosol Med Pulm Drug Deliv.

[26] Yu H, Wang Y, Liu E, Zhang G, Huang L, Liu L (2020). Experimental study of the influences of temperature on the properties of particles in a gasifier during coal-water slurry gasification. ACS Omega.

[27] Oumabady S, Sebastein PS, Kamaludeen SPB, Ramasamy M, Kalaiselvi P, Parameswari E (2020). Preparation and characterization of optimized hydrochar from paper board mill sludge. Sci Rep.

[28] Auffan M, Rose J, Bottero JY, Lowry GV, Jolivet JP, Wiesner MR (2009). Towards a definition of inorganic nanoparticles from an environmental, health and safety perspective. Nat Nanotechnol.

[29] Pietrofesa RA, Park K, Mishra OP, Johnson-McDaniel D, Myerson JW, Shuvaev VV (2021). Copper oxide nanoparticle-induced acute inflammatory response and injury in murine lung is ameliorated by synthetic secoisolariciresinol diglucoside (LGM2605). Int J Mol Sci.

[30] Nishiumi F, Kawai Y, Nakura Y, Yoshimura M, Wu HN, Hamaguchi M (2021). Blockade of endoplasmic reticulum stress-induced cell death by Ureaplasma parvum vacuolating factor. Cell Microbiol.

[31] Sharma S, Ghufran SM, Ghose S, Biswas S (2021). Cytoplasmic vacuolation with endoplasmic reticulum stress directs sorafenib induced non-apoptotic cell death in hepatic stellate cells. Sci Rep.

[32] Li W, Cao T, Luo C, Cai J, Zhou X, Xiao X (2020). Crosstalk between ER stress, NLRP3 inflammasome, and inflammation. Appl Microbiol Biotechnol.

[33] Amen OM, Sarker SD, Ghildyal R, Arya A (2019). Endoplasmic reticulum stress activates unfolded protein response signaling and mediates inflammation, obesity, and cardiac dysfunction: therapeutic and molecular approach. Front Pharmacol.

[34] Laudisi F, Di Grazia A, De Simone V, Cherubini F, Colantoni A, Ortenzi A (2019). Induction of endoplasmic reticulum stress and inhibition of colon carcinogenesis by the anti-helmintic drug rafoxanide. Cancer Lett.

[35] Pardo M, Katra I, Schaeur JJ, Rudich Y (2017). Mitochondria-mediated oxidative stress induced by desert dust in rat alveolar macrophages. Geohealth.

[36] Calderón-Garcidueñas L, Stommel EW, Rajkumar RP, Mukherjee PS, Ayala A (2021). Particulate air pollution and risk of neuropsychiatric outcomes. What we breathe, swallow, and put on our skin matters. Int J Environ Res Public Health.

[37] Liu Y, Lu F, Kang L, Wang Z, Wang Y (2017). Pirfenidone attenuates bleomycin-induced pulmonary fibrosis in mice by regulating Nrf2/Bach1 equilibrium. BMC Pulm Med.

[38] Chillappagari S, Garapati V, Mahavadi P, Naehrlich L, Schmeck BT, Schmitz ML (2021). Defective BACH1/HO-1 regulatory circuits in cystic fibrosis bronchial epithelial cells. J Cyst Fibros.

[39] Leonard R, Zulfikar R, Stansbury R (2020). Coal mining and lung disease in the 21st century. Curr Opin Pulm Med.

[40] Blackley DJ, Reynolds LE, Short C, Carson R, Storey E, Halldin CN (2018). Progressive massive fibrosis in coal miners from 3 clinics in virginia. JAMA.

[41] Hariri LP, Adams DC, Applegate MB, Miller AJ, Roop BW, Villiger M (2019). Distinguishing tumor from associated fibrosis to increase diagnostic biopsy yield with polarization-sensitive optical coherence tomography. Clin Cancer Res.

