# Supplementary material for: Differences in the characteristics and pulmonary toxicity of nano- and micron-sized respirable coal dust
Source: Respir Res. 2022 Jul 30;23:197. doi: 10.1186/s12931-022-02120-8 (PMC9338665; doi:10.1186/s12931-022-02120-8)
Supplement: Supplementary file 1 — Additional file 1: Table S1. Particle size distribution of CD-MPs and CD-NPs by volume. Fig. S1. Chemical and physical properties of the measured coal samples. A. The Zeta potential of the CD-MPs and CD-NPs were measured with a Malvern Nanoparticle Size Potentiometer. B. ESR/EPR was used to investigate the oxygen radicals of CD-MPs and CD-NPs. C. The functional groups of CD-MPs and CD-NPs were analyzed by infrared spectrometer. CD-MPs, coal dust micron particles; CD-NPs, coal dust nanoparticles; ESR, electron spin resonance; EPR, electron paramagnetic resonance. Fig. S2. CD-NPs-induced passage 40 cells were constructed as an in vitro fibrosis model. A. The cell migration was detected with a scratch healing assay. Data were expressed as the mean ± SD, n = 3. *P < 0.05, **P < 0.01 and ***P < 0.001. B. Cell proliferation was detected with CCK-8 assay. Data were expressed as the mean ± SD, n = 3. C. Cell invasion was detected with Transwell assay. Data were expressed as the mean ± SD, n = 3. *P < 0.05, **P < 0.01 and ***P < 0.001. D. The levels of EMT and pro-fibrogenesis marker molecules were detected by western blot. Remarks: Due to insufficient experimental funds, in the western blot assay, we cut the PVDF membrane into a membrane small enough to permit incubation of the antibody according to its molecular weight and the protein molecular weight marker. CD-MPs, coal dust micron particles; CD-NPs, coal dust nanoparticles; EMT, epithelial–mesenchymal transition; P0, passage 0 cells; P10, passage 10 cells; P20, passage 20 cells; P40, passage 40 cells. Table S2. Differences in the characteristics and pulmonary toxicity of nano- and micron-sized respirable coal dust (summary of data and effects). [file 12931_2022_2120_MOESM1_ESM.docx]

Table S1. Particle size distribution of CD-MPs and CD-NPs by volume

| CD-MPs | | CD-NPs | |
| --- | --- | --- | --- |
| Size classes (nm) | Volume distribution data (%) | Size classes (nm) | Volume distribution data (%) |
| 1483.893066 | 0 | 190.1371307 | 0 |
| 1718.465698 | 0.011884841 | 220.1938629 | 0.031297563 |
| 1990.119385 | 0.138999999 | 255.0019379 | 0.621518373 |
| 2304.71582 | 1.417214314 | 295.312439 | 4.15139287 |
| 2669.043457 | 5.745591482 | 341.9951782 | 11.95759429 |
| 3090.963867 | 13.45580355 | 396.0575256 | 19.71586683 |
| 3579.580811 | 21.82997004 | 458.6659546 | 22.43439399 |
| 4145.437988 | 25.25060018 | 531.1715088 | 19.67428234 |
| 4800.745605 | 18.00862036 | 615.1386719 | 13.21381202 |
| 5559.643555 | 11.86473099 | 712.3793335 | 6.183233526 |
| 6438.507813 | 2.276764472 | 824.9916992 | 1.772681342 |
| 7456.301758 | 0 | 955.4057007 | 0.243927591 |
| 8634.988281 | 0 | 1106.435425 | 0 |
| < 5000 | 85.85868477 | < 500 | 58.91206392 |

CD-MPs, coal dust micron particles; CD-NPs, coal dust nanoparticles.


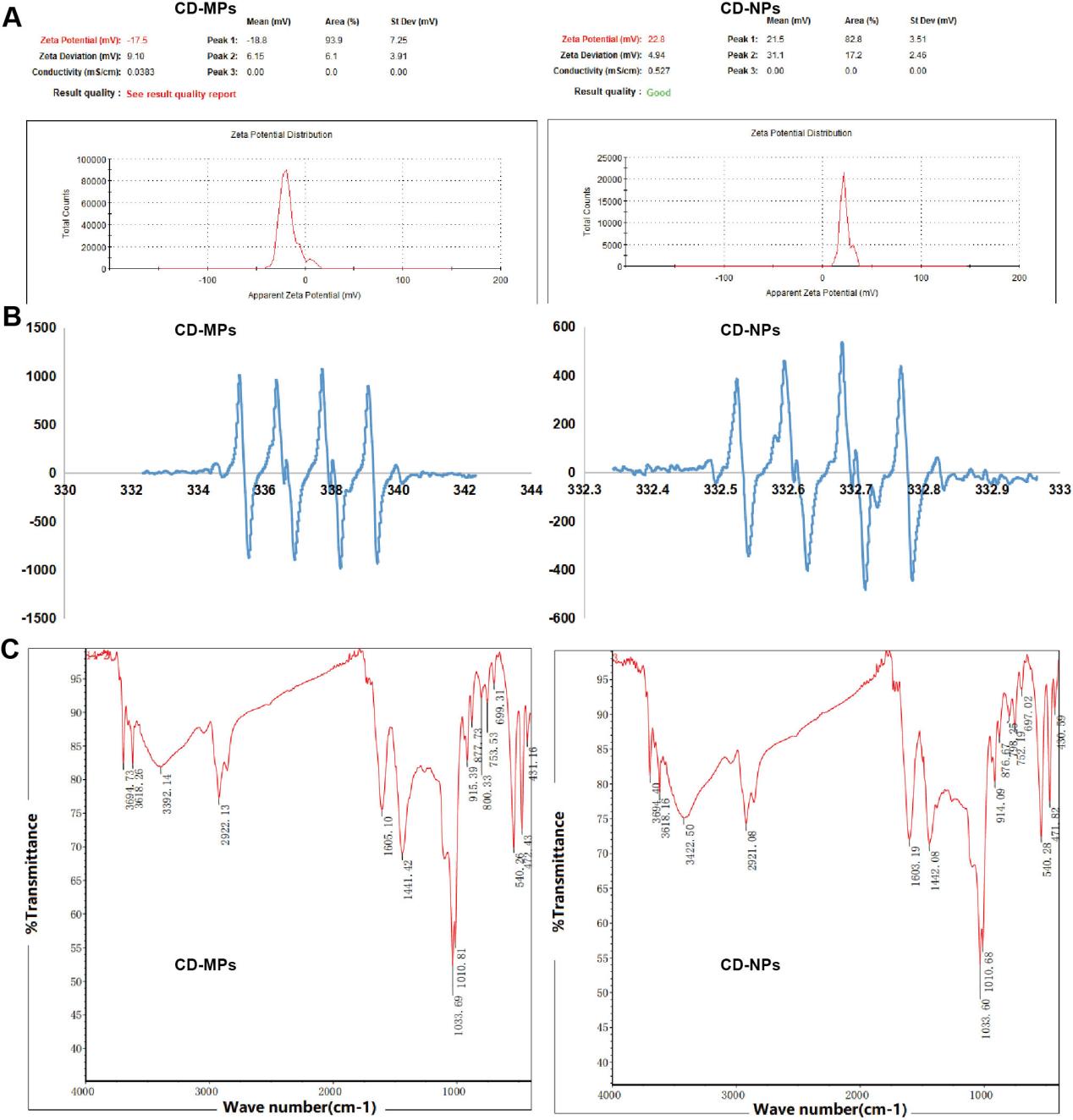


**Fig. S1.** Chemical and physical properties of the measured coal samples. **A.** The Zeta potential of the CD-MPs and CD-NPs were measured with a Malvern Nanoparticle Size Potentiometer. **B.** ESR/EPR was used to investigate the oxygen radicals of CD-MPs and CD-NPs. **C** The functional groups of CD-MPs and CD-NPs were analyzed by infrared spectrometer. CD-MPs, coal dust micron particles; CD-NPs, coal dust nanoparticles; ESR, electron spin resonance; EPR, electron paramagnetic resonance.


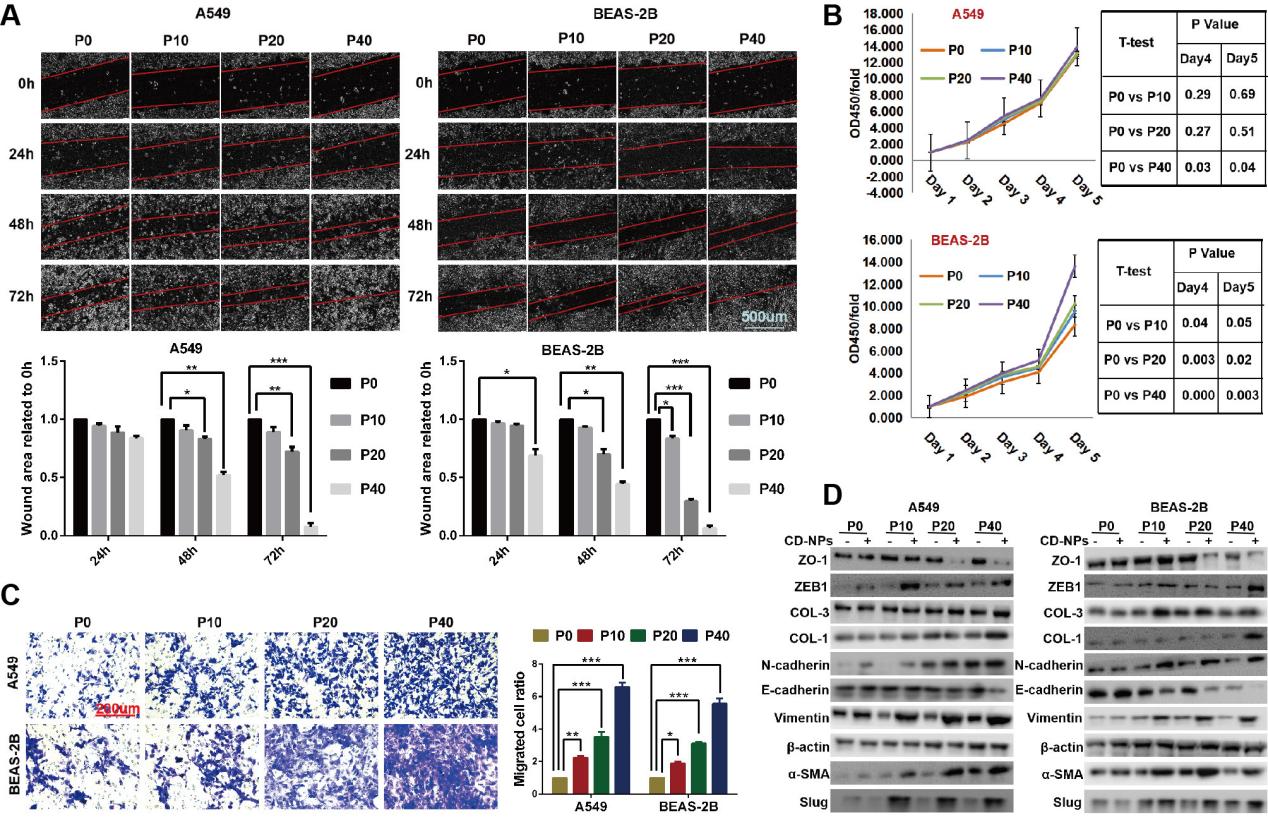


**Fig. S2.**  CD-NPs-induced passage 40 cells were constructed as an in vitro fibrosis model. **A.** The cell migration was detected with a scratch healing assay. Data were expressed as the mean ± SD, n=3. *P < .05, **P < .01 and ***P < .001. **B.** Cell proliferation was detected with CCK-8 assay. Data were expressed as the mean ± SD, n=3. **C.**  Cell invasion was detected with Transwell assay. Data were expressed as the mean ± SD, n=3. *P < .05, **P < .01 and ***P < .001. **D.** The levels of EMT and pro-fibrogenesis marker molecules were detected by western blot. Remarks: Due to insufficient experimental funds, in the western blot assay, we cut the PVDF membrane into a membrane small enough to permit incubation of the antibody according to its molecular weight and the protein molecular weight marker. CD-MPs, coal dust micron particles; CD-NPs, coal dust nanoparticles; EMT, epithelial-mesenchymal transition; P0, passage 0 cells; P10, passage 10 cells; P20, passage 20 cells; P40, passage 40 cells.

Table S2. Differences in the characteristics and pulmonary toxicity of nano- and micron-sized respirable coal dust (summary of data and effects)

|  |  | | | | | CD-MPs | CD-NPs | CD-MPs vs CD-NPs |
| --- | --- | --- | --- | --- | --- | --- | --- | --- |
| Characteristics | Morphology | | | | | Irregular particles | Tended to be regular spherical particles |  |
|  | Size distribution | | | | | <5 μm (86%) | <500 nm (59%) |  |
|  | Elemental composition | | | | | C: 54% (wt); O: 27% (wt); Al: 9% (wt); Si: 9% (wt) | C: 75% (wt); O: 17% (wt); Al: 2% (wt); Si: 2% (wt) |  |
|  | Zeta potential | | | | | -17.5 mV | 22.8 mV |  |
|  | Oxygen radicals | | | | | Superoxide radical (DMPO-O2·- ) | Superoxide radical (DMPO-O2·- ) |  |
|  | Functional groups | | | | | -OH; C-O; C-I; C-Br; O-H | -OH; C-O; C-I; C-Br; O-H |  |
| In vitro effects | Acute toxic effects | A549 | Cell viability (48 h: IC50 (μg/mL)) | | | 727.768 | 228.065 | *** |
|  |  |  | Inflammatory vacuoles | | | Few, small, scattered | Numerous, large, dense |  |
|  |  |  | Profibrotic cytokines levels (Mean) | CXCL2 (ng/mL) | | 2.37 | 4.07 | *** |
|  |  |  |  | TGFβ1 (pg/mL) | | 57.40 | 88.43 | ** |
|  |  |  | Pro-inflammatory cytokines levels (Mean) | IL-6 (pg/mL) | | 19.77 | 31.07 | *** |
|  |  |  |  | IL-1β (pg/mL) | | 55.53 | 122.23 | *** |
|  |  |  |  | TNFα (pg/mL) | | 25.10 | 32.13 | * |
|  |  |  | Mitochondrial damage (Mean) | Mitochondrial membrane potential (JC-1 ratio) | | 0.16 | 0.03 | *** |
|  |  |  |  | ROS levels (Fluorescence intensity ratio) | | 1.89 (8 h) | 5.52 (8 h) | *** |
|  |  |  |  | Ca^2+^ levels (Fluorescence intensity ratio) | | 2.32 (8 h) | 5.58 (8 h) | *** |
|  |  |  |  | HO-1/β-actin protein level ratio | | 0.83 (8 h) | 1.12 (8 h) | ** |
|  |  |  | Cell death (Mean) | Cleaved Caspase1/Caspase1 protein level ratio | | 1.31 | 3.78 | *** |
|  |  |  |  | Necrotic cells (%) | AnnxinV-FITC/PI staining | 48 | 57 | * |
|  |  |  |  |  | AO/EB staining | 19 | 43 | ** |
|  |  |  |  | Bcl-2/β-actin protein level ratio | | 1.47 | 1.26 | * |
|  |  | BEAS-2B | Cell viability (48 h: IC50 (μg/mL)) | | | 1302.739 | 686.776 | *** |
|  |  |  | Inflammatory vacuoles | | | Few, small, scattered | Numerous, large, dense |  |
|  |  |  | Profibrotic cytokines levels (Mean) | CXCL2 (ng/mL) | | 3.05 | 5.06 | *** |
|  |  |  |  | TGFβ1 (pg/mL) | | 46.47 | 73.70 | ** |
|  |  |  | Pro-inflammatory cytokines levels (Mean) | IL-6 (pg/mL) | | 16.16 | 23.43 | * |
|  |  |  |  | IL-1β (pg/mL) | | 64.60 | 119.13 | ** |
|  |  |  |  | TNFα (pg/mL) | | 23.20 | 29.07 | * |
|  |  |  | Mitochondrial damage (Mean) | Mitochondrial membrane potential (JC-1 ratio) | | 0.37 | 0.05 | *** |
|  |  |  |  | ROS levels (Fluorescence intensity ratio) | | 1.82 (8 h) | 4.64 (8 h) | *** |
|  |  |  |  | Ca^2+^ levels (Fluorescence intensity ratio) | | 1.24 (8 h) | 2.28 (8 h) | ** |
|  |  |  |  | HO-1/β-actin protein level ratio | | 0.98 (8 h) | 1.45 (8 h) | *** |
|  |  |  | Cell death (Mean) | Cleaved Caspase1/Caspase1 protein level ratio | | 0.87 | 2.18 | *** |
|  |  |  |  | Necrotic cells (%) | AnnxinV-FITC/PI staining | 7 | 13 | ** |
|  |  |  |  |  | AO/EB staining | 8 | 15 | ** |
|  |  |  |  | Bcl-2/β-actin protein level ratio | | 1.36 | 1.40 | n.s |
|  | Chronic toxic effects | A549 | Cell proliferation ability (Mean: OD450/fold-day 5) | | | 11.641 | 13.715 | ** |
|  |  |  | Cell migration ability (Mean: Wound area related to 0 h) | | | 0.65 | 0.39 | *** |
|  |  |  | Cell invasive ability (Mean: Migrated cell ratio) | | | 3.61 | 5.50 | *** |
|  |  |  | EMT marker molecule/β-actin protein level ratio (Mean) | ZO-1 | | 0.92 | 0.21 | *** |
|  |  |  |  | ZEB1 | | 0.37 | 1.04 | *** |
|  |  |  |  | N-cadherin | | 0.74 | 1.04 | ** |
|  |  |  |  | E-cadherin | | 1.05 | 0.38 | ** |
|  |  |  |  | Vimentin | | 1.22 | 1.39 | * |
|  |  |  |  | Slug | | 0.62 | 0.94 | * |
|  |  |  | Profibrotic marker molecule/β-actin protein level ratio (Mean) | COL-1 | | 0.31 | 0.53 | * |
|  |  |  |  | COL-3 | | 1.16 | 1.39 | * |
|  |  |  |  | α-SMA | | 0.12 | 0.70 | ** |
|  |  | BEAS-2B | Cell proliferation ability (Mean: OD450/fold-day 5) | | | 8.948 | 10.724 | *** |
|  |  |  | Cell migration ability (Mean: Wound area related to 0 h) | | | 0.72 | 0.40 | *** |
|  |  |  | Cell invasive ability (Mean: Migrated cell ratio) | | | 3.99 | 5.24 | *** |
|  |  |  | EMT marker molecule/β-actin protein level ratio (Mean) | ZO-1 | | 1.41 | 0.91 | *** |
|  |  |  |  | ZEB1 | | 0.60 | 1.44 | *** |
|  |  |  |  | N-cadherin | | 1.22 | 1.61 | ** |
|  |  |  |  | E-cadherin | | 1.34 | 1.05 | * |
|  |  |  |  | Vimentin | | 1.38 | 1.77 | * |
|  |  |  |  | Slug | | 1.25 | 1.43 | * |
|  |  |  | Profibrotic marker molecule/β-actin protein level ratio (Mean) | COL-1 | | 1.18 | 2.39 | *** |
|  |  |  |  | COL-3 | | 1.22 | 1.61 | ** |
|  |  |  |  | α-SMA | | 1.00 | 2.22 | *** |
| In vivo effects | Acute toxic effects | Alveolar inflammation score (Mean)  The degree of alveolitis  was evaluated according to the scoring  system of Szapiel et al. (1979).^1^ | | | | 1.33 | 2.67 | * |
|  |  | BALF | Profibrotic cytokines levels (Mean) | CXCL2 (pg/mL) | | 120.10 | 193.33 | *** |
|  |  |  |  | TGFβ1 (pg/mL) | | 31.73 | 83.57 | *** |
|  |  |  | Pro-inflammatory cytokines levels (Mean) | IL-6 (pg/mL) | | 37.77 | 136.87 | *** |
|  |  |  |  | IL-1β (pg/mL) | | 97.93 | 271.93 | *** |
|  |  |  |  | TNFα (pg/mL) | | 61.20 | 95.70 | ** |
|  | Chronic toxic effects | Morphology of lung tissue | | | | Fine coal spots, slightly off-white, slightly hard texture | Sparse coal spots, slightly off-white, slightly hard texture |  |
|  |  | Lung organ coefficient (%)-Mean | | | | 1.1 | 1.3 | * |
|  |  | Lung tissue fibrosis score (Mean)  The degree of pulmonary fibrosis was evaluated according to the scoring  system of Szapiel et al. (1979).^1^ | | | | 1.33 | 2.67 | * |
|  |  | EMT marker molecule/β-actin protein level ratio (Mean) | | ZO-1 | | 1.19 | 0.28 | *** |
|  |  |  |  | ZEB1 | | 0.19 | 0.53 | ** |
|  |  |  |  | N-cadherin | | 0.96 | 1.19 | * |
|  |  |  |  | E-cadherin | | 1.03 | 0.78 | * |
|  |  |  |  | Vimentin | | 1.05 | 1.25 | * |
|  |  |  |  | Slug | | 0.33 | 0.53 | * |
|  |  | Profibrotic marker molecule/β-actin protein level ratio (Mean) | | COL-1 | | 0.36 | 1.61 | *** |
|  |  |  |  | COL-3 | | 1.21 | 1.35 | * |
|  |  |  |  | α-SMA | | 0.65 | 1.13 | ** |
|  |  | BALF | Profibrotic cytokines levels (Mean) | CXCL2 (pg/mL) | | 118.67 | 188.60 | *** |
|  |  |  |  | TGFβ1 (pg/mL) | | 31.63 | 84.63 | *** |
|  |  |  | Pro-inflammatory cytokines levels (BALF) | IL-6 (pg/mL) | | 35.27 | 123.40 | *** |
|  |  |  |  | IL-1β (pg/mL) | | 94.63 | 246.03 | *** |
|  |  |  |  | TNFα (pg/mL) | | 56.37 | 90.47 | ** |
|  |  | Serum | Profibrotic cytokines levels (Mean) | CXCL2 (ng/mL) | | 65.73 | 103.83 | ** |
|  |  |  |  | TGFβ1 (pg/mL) | | 20.73 | 44.77 | ** |
|  |  |  | Pro-inflammatory cytokines levels (BALF) | IL-6 (pg/mL) | | 27.03 | 70.40 | ** |
|  |  |  |  | IL-1β (pg/mL) | | 81.73 | 156.47 | *** |
|  |  |  |  | TNFα (pg/mL) | | 50.23 | 80.33 | *** |

CD-MPs, coal dust micron particles; CD-NPs, coal dust nanoparticles; C, carbon; O, oxygen; Al, aluminum; Si, silicon; vs, versus; wt:, weight; IC50, half maximal inhibitory concentration; ROS, reactive oxygen species; h. hour; HO-1,heme oxygenase-1; EMT, epithelial-mesenchymal transition; BALF, bronchoalveolar lavage fluid; *P < 0.05, **P < 0.01, ***P < 0.001.

1. Szapiel, S. V., Elson, N. A., Fulmer, J. D., Hunninghake, G. W., and Crystal, R. G. (1979). Bleomycin-induced Interstitial Pulmonary Disease in the Nude, Athymic Mouse. Am. Rev. Respir. Dis. 120 (4), 893–899. doi:10.1164/arrd.1979.120.4.893

The degree of alveolitis and pulmonary fibrosis was evaluated according to the scoring system outlined in Szapiel et al. (1979). Alveolitis was graded using the following criteria: None (0), no alveolitis; mild (1+), thickening of the alveolar septum by a mononuclear cell infiltrate; moderate (2+), a more widespread alveolitis; severe (3+), a diffuse alveolitis. The extent of fibrosis was graded using the following criteria: none (0), no fibrosis; mild (1+), focal regions of fibrosis, alveolar architecture has some distortion; moderate (2+), more extensive fibrosis and fibrotic still focal; severe (3+), widespread fibrosis, confluent lesions with extensive derangement of parenchymal architecture.
